# TDP-43 regulates site-specific 2′-*O*-methylation of U1 and U2 snRNAs via controlling the Cajal body localization of a subset of C/D scaRNAs

**DOI:** 10.1093/nar/gkz086

**Published:** 2019-02-13

**Authors:** Keiichi Izumikawa, Yuko Nobe, Hideaki Ishikawa, Yoshio Yamauchi, Masato Taoka, Ko Sato, Hiroshi Nakayama, Richard J Simpson, Toshiaki Isobe, Nobuhiro Takahashi

**Affiliations:** 1 Department of Applied Biological Science and Global Innovation Research Organizations, Tokyo University of Agriculture and Technology, 3-5-8 Saiwai-cho, Fuchu, Tokyo 183–8509, Japan; 2 Department of Chemistry, Graduate School of Science, Tokyo Metropolitan University, 1-1 Minami-ohsawa, Hachioji, Tokyo 192–0397, Japan; 3 Biomolecular Characterization Unit, RIKEN Center for Sustainable Resource Science, 2-1, Hirosawa, Wako, Saitama 351-0198, Japan; 4 La Trobe Institute for Molecular Science (LIMS), LIMS Building 1, Room 412 La Trobe University, Melbourne Victoria 3086, Australia

## Abstract

TDP-43 regulates cellular levels of Cajal bodies (CBs) that provide platforms for the assembly and RNA modifications of small nuclear ribonucleoproteins (snRNPs) involved in pre-mRNA splicing. Alterations in these snRNPs may be linked to pathogenesis of amyotrophic lateral sclerosis. However, specific roles for TDP-43 in CBs remain unknown. Here, we demonstrate that TDP-43 regulates the CB localization of four UG-rich motif-bearing C/D-box-containing small Cajal body-specific RNAs (C/D scaRNAs; i.e. scaRNA2, 7, 9 and 28) through the direct binding to these scaRNAs. TDP-43 enhances binding of a CB-localizing protein, WD40-repeat protein 79 (WDR79), to a subpopulation of scaRNA2 and scaRNA28; the remaining population of the four C/D scaRNAs was localized to CB-like structures even with WDR79 depletion. Depletion of TDP-43, in contrast, shifted the localization of these C/D scaRNAs, mainly into the nucleolus, as well as destabilizing scaRNA2, and reduced the site-specific 2′-*O*-methylation of U1 and U2 snRNAs, including at 70A in U1 snRNA and, 19G, 25G, 47U and 61C in U2 snRNA. Collectively, we suggest that TDP-43 and WDR79 have separate roles in determining CB localization of subsets of C/D and H/ACA scaRNAs.

## INTRODUCTION

The trans-activating response region DNA-binding protein 43 (TARDBP, TDP-43) is a product of a causative gene for amyotrophic lateral sclerosis (ALS), and its disease-associated mutations cause its accumulation in the cytoplasm of cells in ALS-related lesions in the brain (1,2). In normal cells, TDP-43 is ubiquitously expressed and is shuttled between the nucleus and cytoplasm but resides mostly in the nucleus at steady state (3). TDP-43 preferentially recognizes UG-rich motifs that are found in thousands of pre-mRNAs (4–6) and auto-regulates its cellular level via binding to the UG-rich motif of its own mRNA by a negative feedback mechanism through which it affects the stability of its mRNA (7,8). Although many cellular functions, including those related to transcription and mRNA transport and in mitochondria, have been reported for TDP-43 (9–12), the best-characterized function of TDP-43 is probably the regulation of pre-mRNA splicing. TDP-43 regulates splicing partly via binding to the *cis*-elements of introns and/or partly via actions with splicing factors (such as serine/arginine-rich proteins) or RNA-binding proteins (such as hnRNP A2) (5,13–17).

TDP-43 conditional-knockout mice and TDP-43 knockdown in cells in culture reduce the number of gems (10,18). Fibroblast cell lines expressing TDP-43 with an ALS-causing mutation originally isolated from ALS patients also have about half the number of gems relative to normal cells (19), indicating that TDP-43 is required for maintaining the cellular level of gems, although the specific mechanism by which it carries out this effect is still unknown. In most human tissues and cells, gems are very similar to Cajal bodies (CBs) in terms of their protein components and of immunocytochemical staining, so CBs and gems are regarded as just two different faces of the same structure (20). Therefore, we will treat gems as CBs from now on unless they are specifically indicated. CBs are multifunctional subnuclear structures that provide platforms for the assembly and RNA modification of small nuclear ribonucleoproteins (snRNPs) involved in pre-mRNA splicing, ribosome biogenesis, histone mRNA processing and telomere synthesis, and their numbers per cell vary among cell types and seem to increase in transcriptionally active cells, such as neuronal cells, embryonic cells, cancer cells etc. (21–25). CBs contain many factors, including coilin (a key protein to form CBs), constituents of SMN (survival motor neuron protein) complex (26,27) and small CB-specific RNAs (scaRNAs) (21,28,29). The SMN complex assists the assembly of snRNPs, mostly in the cytoplasm, whereas the scaRNAs guide site-specific post-transcriptional modifications including pseudouridylation and 2′-*O*-methylation of RNA components of snRNPs in CBs, which lead to the last step of the assembly of snRNPs with specific RNP proteins (21,28–31). In CBs, scaRNAs having a box H/ACA motif (H/ACA scaRNAs) guide pseudouridylation of snRNA with four core proteins (dyskerin, Nop10, Nhp2 and Gar1), whereas scaRNAs having the box C/D motif (C/D scaRNAs) guide 2′-*O*-methylation of snRNAs with the other four core proteins (fibrillarin, 15.5-kDa protein, Nop56 and Nop58) (32,33). The CB localization of H/ACA scaRNAs is determined by their conserved signal sequence (the CAB box) located in the terminal stem–loop of the scaRNAs (34). This CAB box binds WDR79 containing a WD40 domain, which drives the CB localization of the target RNAs (35,36). In contrast, the CB localization of a subset of C/D scaRNAs is determined by the UG-rich motif that forms consecutive G.U and U.G wobble base-pairs with the terminal stem–loop of the RNA apical hairpin. Instead of the CAB box present in the H/ACA scaRNAs, the G.U and U.G wobble base-pairs of C/D scaRNAs are essential for the CB-specific localization of C/D scaRNAs and are required for the binding to WDR79 (36). However, WDR79 binds C/D scaRNAs several-fold less efficiently than do H/ACA scaRNAs (32,33,35), such that involvement of other unknown factor(s) in the CB localization of C/D scaRNAs has been proposed (32,33,36). In this study, we identified TDP-43 as a major player that regulates the CB localization of a subset of C/D scaRNAs independently of WDR79.

## MATERIALS AND METHODS

### Antibodies and reagents

Antibodies used in this study are listed in Supplementary Table S1. Stabilized Streptavidin-HRP Conjugate (Thermo Fischer Scientific, 89880D) was used for the detection of biotinylated oligonucleotides or proteins. All general reagents were purchased from Wako Pure Chemical (Osaka, Japan), Kanto Chemical Co. (Tokyo, Japan) or Nacalai Tesque (Kyoto, Japan).

### Cell culture

HeLa, 293T (HEK293 cells transformed with large T antigen) and Flp-In T-REx 293 (T-REx 293) cells (Thermo Fisher Scientific) were cultured in Dulbecco’s modified Eagle’s medium (DMEM; Sigma-Aldrich) supplemented with 10% heat-inactivated fetal bovine serum (Biowest LLC) and streptomycin (0.1 mg/ml; Wako) and penicillin G (100 U/ml, Wako) at 37°C under 5% CO_2_ in air.

### Construction of epitope-tagged expression plasmids

The construction of the plasmids expressing DAP (His6-, biotin- and FLAG-tagged)-TDP-43 (DAP-TDP-43 pcDNA5FRT/TO, wt) and TDP-43 deletion mutants (Δ315, ΔGR, ΔRRM2, ΔRRM1) was described previously (12). To construct the plasmid expressing HEF (HA, TEV cleavage site and FLAG)-tagged WDR79 (HEF-WDR79 pcDNA5FRT/TO), DNA fragment encoding WDR79 was amplified by KOD-Plus-neo (TOYOBO, Japan) with a primer set (WDR79-for/WDR79-rev) using cDNA derived from 293T as a template DNA. The amplified DNA was digested with HindIII and XhoI, and inserted into the HindIII/ XhoI site of HEF-pcDNA5FRT/TO constructed as described (37). To construct a plasmid expressing scaRNA28, a DNA fragment covering a region from exon2 to exon3 of TRRAP was amplified from genomic DNA prepared from 293T cells as a template DNA by using KOD-Plus-neo with a primer set (TRRAP-for/TRRAP-rev). The amplified DNA was digested with BamHI/ XhoI and inserted into the BamHI/ XhoI site of DAP-pcDNA5FRT/TO, generating the final construct DAP-TRRAP(ex2-3)-pcDNA5FRT/TO. All sequences of the resulting plasmids were confirmed by DNA sequencing. The construction of DAP-LYAR-pcDNA5FRT/TO (or HBF-LYAR-pcDNA5FRT/TO) was done as described previously (38). FLAG-TDP-12xQ/N-pcDNA5FRT/TO was provided by Dr. Emanuele Buratti in the International Centre for Genetic Engineering and Biotechnology (ICGEB), Trieste, Italy. Primer sequences used for PCR are listed in Supplementary Table S2.

### Construction of doxycycline-inducible cell lines

T-REx 293 cell lines inducibly expressing protein with doxycycline were established as described previously (12,39). To express a protein, 100 ng/ml of doxycycline was treated to the cells for the indicated time periods in the corresponding figure legends.

### Pull-down of TDP-43−binding RNAs for LC-MS analysis

DAP-TDP-43-expressing T-REx 293 cells (induced with 100 ng/ml of doxycycline for 24 h) were harvested, washed twice with ice-cold PBS, suspended in extraction buffer (67 mM Tris–HCl, pH 7.4; 200 mM NaCl; 0.1% IGEPAL CA-630 (SIGMA); 1 mM phenylmethylsulfonyl fluoride (PMSF)) with 1 mM Ribonucleoside–Vanadyl Complex (New England BioLabs) and lysed by sonication using a Bioruptor 200 (highest setting, six 30-s pulses with 30-s intervals, 4°C; CosmoBio, Japan). After centrifugation at 20 000 *g* for 10 min at 4°C, the supernatant was collected as the total cell extract. For the first step pull-down, the extract (10 mg) was incubated with 30 μl of Ni-NTA Agarose (Qiagen) by rotation for 1 h at 4°C. The DAP-TDP-43 bound Agarose was washed five times with wash buffer (67 mM Tris–HCl, pH 7.4; 200 mM NaCl; 0.1% IGEPAL CA-630) and eluted with 100 μl of imidazole-containing buffer (67 mM Tris–HCl, pH 7.4; 200 mM NaCl; 0.1% IGEPAL CA-630; 250 mM imidazole) for 10 min twice on ice. The eluates recovered as His6-tagged protein complexes were diluted with 400 μl of wash buffer containing 1 mM PMSF and incubated with 15 μl FLAG M2 Agarose beads (Sigma-Aldrich) for 4 h at 4°C for the second step pull-down. After the beads were washed five times with wash buffer, the DAP-TDP-43 complexes were eluted with 150 μl of Protein-RNA extraction buffer (7 M urea; 350 mM NaCl; 1% SDS; 10 mM Tris–HCl, pH 8.0; 10 mM ethylenediaminetetraacetic acid (EDTA); 2% 2-mercaptoethanol) for 5 min at 25°C as FLAG-tagged complexes. The purified DAP-TDP-43 complexes were subjected to phenol–chloroform extraction; namely, 150 μl of eluate was added to 100 μl of phenol–chloroform (pH 8.0) (Thermo Fisher Scientific) and 15 μl of 3 M sodium acetate (pH 5.2). After vigorous mixing for 10 min at room temperature, the organic phase containing protein components and the aqueous phase containing RNA components were separated by centrifugation at 20 000 *g* for 10 min at 4°C. The separately precipitated proteins and RNAs by isopropanol precipitation were washed with 75% ethanol and dried. Proteins were analyzed by immunoblotting and RNAs were suspended in 100% formamide and separated on a 6% (19:1 acrylamide/bis-acrylamide) denaturing urea polyacrylamide gel with 0.5× TBE buffer. The gel was stained with SYBR Gold (Thermo Fisher Scientific) and visualized by LAS-4000 (GE healthcare).

### In-gel RNA digestion and LC-MS analysis for RNA identification

In-gel RNA digestion and LC-MS analysis for RNA identification were performed as described (40). Assignment of MS/MS spectra and RNA identification were done by using Ariadne (41) (http://ariadne.riken.jp/) with the human genome database (GRCh37.p5) or a human small RNA database containing all sequences of the transcripts registered in RefSeq (29 May 2018, shorter than 1000 bases) plus those of 18S rRNA (NR_145820) and 28S rRNA (NR_145822). The following default search parameters for Ariadne were used: maximum number of missed cleavages, 1; variable modification parameters, one methylation per RNA fragment for any residue; RNA mass tolerance, ±5 ppm, and MS/MS tolerance, ±20 ppm.

### Immunoprecipitation with FLAG-tagged protein

T-REx 293 cells expressing a FLAG-tagged protein inducibly were harvested, washed twice with ice cold PBS, suspended with extraction buffer (67 mM Tris–HCl, pH 7.4, 200 mM NaCl, 0.1% IGEPAL CA-630 (SIGMA), 1 mM Ribonucleoside–Vanadyl Complex (New England BioLabs), 1 mM PMSF) and lysed by sonication using Bioruptor 200 (highest setting, six times for 30 s, 4°C; CosmoBio, Japan). After centrifugation at 20 000 *g* for 10 min at 4°C, the supernatant was rotate-incubated with 15 μl of FLAG M2 Agarose beads (Sigma-Aldrich) for 2 h at 4°C. The Agarose beads were washed five times with wash buffer (67 mM Tris–HCl, pH 7.4, 200 mM NaCl, 0.1% IGEPAL CA-630) and eluted for recovery of FLAG-tagged protein complexes with 150 μl of Protein-RNA extraction buffer (7 M urea, 350 mM NaCl, 1% SDS, 10 mM Tris–HCl, pH 8.0, 10 mM EDTA and 2% 2-mercaptoethanol) for 5 min at 25°C. Protein or RNA components were extracted as described in above section ‘Pull down of TDP-43-binding RNA for LC-MS analysis’, and subjected to the immunoblot or northern blot analysis as described below.

### Immunoblot analysis

Immunoblot analysis was performed as described (39). Protein bands were detected using LAS-4000 system (GE healthcare). Antibodies used in this study were listed in Supplementary Table S1.

### Northern blot analysis

Northern blot analysis was performed as described (12). The biotin-labeled DNA probes used were listed in Supplementary Table S2.

### UV-CLIP

DAP-TDP-43-expressed T-REx 293 cells were cultured in a 150-mm Petri dish, exposed to UV light at 400 mJ/cm^2^ for cross-linking and harvested with ice-cold PBS. After suspended in 1 ml of extraction buffer and 175 μl of 2.5 M NaCl (0.5 M NaCl final concentration) on ice for 10 min, the harvested cells were centrifuged at 20 000 *g* for 10 min at 4°C and used for subsequent immunoprecipitation. To isolate DAP-TDP-43 complexes, 15 μl of FLAG M2 Agarose beads were incubated for 3 h with rotation at 4°C, washed five times with wash buffer (50 mM Tris–HCl, pH 7.4; 500 mM NaCl; 0.1% IGEPAL CA-630) and eluted with sodium dodecyl sulphate-polyacrylamide gel electrophoresis (SDS-PAGE) sample buffer after incubation for 3 min at 65°C to recover DAP-TDP-43 complexes. The proteins were separated by SDS-PAGE on a 7% acrylamide gel and electrophoretically transferred to a nitrocellulose membrane (Protran BA 85, 0.45 μm, Whatman). RNAs cross-linked with DAP-TDP-43 were detected using streptavidin-HRP and visualized by LAS-4000. RNAs sifted by cross-linking with TDP-43 were excised from the membrane, treated with 2 mg/ml proteinase K (Takara Bio, Japan) in PK buffer (10 mM Tris–HCl, pH 7.4; 30 mM NaCl; 10 mM EDTA) for 30 min at 37°C, mixed with 250 μl of Protein-RNA extraction buffer, and extracted with phenol–chloroform and then precipitated with isopropanol. RNAs were separated using denaturing urea PAGE with a 6% acrylamide gel and subjected to northern blot analysis.

### scaRNA synthesis by *in vitro* transcription

To synthesize biotin-labeled scaRNAs, using KOD–plus-neo DNA polymerase (Toyobo, Tokyo, Japan), we first amplified DNA fragments containing a T7 promoter sequence at their 5′ ends and scaRNA2, scaRNA7, scaRNA9 or scaRNA28 at 3′ side from their corresponding template DNAs; scaRNA2-pcDNA3.1 (42), pCMV-globin-U90 (36), scaRNA9-pcDNA3.1 (43) or DAP-TRRAP(ex2-3)-pcDNA5FRT/TO (scaRNA28), by using primer sets scaRNA2-Fw/scaRNA2-Rv, scaRNA7-Fw/scaRNA7-Rv, scaRNA9-Fw/scaRNA-Rv or scaRNA28-Fw/scaRNA28-Rv, respectively. The amplified DNA was purified using QIAquick PCR purification kit (Qiagen) and used for *in vitro* transcription of biotin-labeled scaRNAs (0.06 pmol**/**reaction) in the presence of 7.5 mM ATP, 7.5 mM CTP, 7.5 mM UTP, 7.5 mM GTP and 0.5 mM biotin-UTP (Roche) using CUGA7 *in vitro* transcription kit (NIPPON GENE, Japan). The transcripts were collected by isopropanol precipitation, separated by denaturing urea PAGE on a 6% acrylamide gel and visualized by SYBR Gold staining. Each of the biotin-labeled scaRNAs was excised from its corresponding gel band, extracted from the gel and subjected to electrophoresis mobility sift assay. Primer sequences used for PCR are listed in Supplementary Table S2.

### Electrophoresis mobility sift assay (EMSA)

Recombinant proteins (TF-FL and TF-TDP-43-FL) were purified and used for EMSA as described previously with some modifications (12). Briefly, RNA and protein were mixed in 10 μl binding buffer (40 mM Tris–HCl, pH 7.4, 30 mM KCl, 1 mM MgCl_2_, 0.01% IGEPAL CA-630 (octylphenoxy poly(ethyleneoxy)ethanol), 1 mM dithiothreitol (DTT), 10 μg of yeast tRNA (Thermo Fisher Scientific), 10 μg bovine serum albumin (BSA), 10 ng of synthetic RNA, 200 ng of recombinant protein) at 25°C for 20 min. RNA–protein complexes were separated by 6% non-denaturing polyacrylamide gel electrophoresis at 100 V for 70 min in 0.5× Tris borate/EDTA buffer (44.5 mM Tris-borate and 1 mM EDTA), and transferred to a Hybond N+ membrane. The membrane was sequentially dried and UV-crosslinked using the CX-2000 UV crosslinker (UVP) at 120 mJ/cm^2^. The biotin-labeled scaRNAs were detected with Stabilized Streptavidine-HRP-conjugate and visualized by LAS-4000.

### Quantitative real-time PCR (RT-qPCR)

Total RNA (200–300 ng) was isolated using TRIzol Reagent (Thermo Fisher Scientific, Waltham, MA, USA) and subjected to reverse transcription with random hexamer and oligo dT primers using PrimeScript RT Reagent kit (TAKARA BIO, Japan). qPCR was performed using GeneAce SYBR qPCR Mix (NIPPON GENE, Japan) and Thermal Cycler Dice Real Time System (TAKARA BIO, Japan). The value of gene expression was calculated by delta-delta Ct method and normalized for that of *GAPDH* mRNA. Primer sequences used for qPCR are listed in Supplementary Table S2.

### RNA interference (RNAi)

Stealth RNA interference (RNAi) siRNA or Silencer Select siRNA (siRNA) and negative control siRNA or non-specific control RNA (ncRNA) were purchased from Thermo Fisher Scientific. Transfection of siRNA or ncRNA was performed using Lipofectamine RNAiMax (Thermo Fisher Scientific). In six-well plate, 100 pmol of Stealth RNAi siRNA/ncRNA or 25 pmol of Silencer Select siRNA/ncRNA was transfected into 293T or T-REx 293 cells, and 50 pmol of Stealth RNAi siRNA/ncRNA or 12.5 pmol of Silencer Select siRNA/ncRNA into HeLa cells. All siRNA sequences are listed in Supplementary Table S2.

### RNA preparation for LC-MS analysis

Total RNA (20–30 μg) was isolated from HeLa or 293T cells cultured in a 90-mm Petri dish by using TRIzol Reagent. Small RNA fraction (containing U1, U2 snRNA and 5.8S rRNA) and large RNA fraction (containing 28S and 18S rRNA) were prepared from 10 μg of total RNA using mirVana miRNA Isolation Kit (Thermo Fisher Scientific AM1560). 28S or 18S rRNA was purified from large RNA fraction by reverse-phase LC as described in (44). Small RNA fraction was separated using 7% acrylamide denaturing urea PAGE and visualized with SYBR gold staining. RNAs corresponding to U1, U2 snRNA or 5.8S rRNA were excised, and eluted from PAGE gel piece by soaking with buffer containing 20 mM sodium acetate (pH 5.2). U1 or U2 snRNA purified from 10 μg of total RNA by reversed-phase LC on a PLRP-S 300Å column (2.1 × 100 mm, 3 μm, Agilent Technologies) was used for sequence-specific RNase H cleavage.

### *In vitro* transcription of RNAs for internal standard of SILNAS

For *in vitro* transcription of human ^13^C-labeled U1 snRNA, a cDNA fragment encoding U1 snRNA was amplified by PCR from random primed HeLa cDNA library and inserted into the EcoRI/XhoI sites of plasmid pBluescript II KS(+) (Agilent Technologies, Inc.). ^13^C-labeled U1 snRNA was transcribed *in vitro* from SpeI-linearized plasmid (1 μg) using Megascript T3 kit (Thermo Fisher Scientific). To transcribe ^13^C-labeled U2 snRNA, a cDNA fragment encoding human U2 snRNA was amplified from random primed TK6 cDNA library by PCR, inserted into HindIII/XhoI sites of pcDNA3.1(+) vector using a GeneArt Seamless Cloning and Assembly Enzyme Mix (Thermo Fisher Scientific) and used as a template DNA for the transcription after linearized with XhoI (1 μg). For RNase T1 digestion, RNA was synthesized using Guanosine-^13^C_10_ 5′-triphosphate, whereas for RNase A digestion, cytidine-^13^C_9_ 5′-triphosphate and uridine-^13^C_9_ 5′-triphosphate were used instead of the respective 5′-triphosphate reagent that contained carbons with natural isotope distribution. The ^13^C-labeled U1 or U2 snRNA was precipitated in ethanol, solubilized in nuclease-free water and then purified further by reversed-phase LC as described above. The ^13^C-labeled 28S, 18S and 5.8S rRNA were synthesized as described (44). Primer sequences used for PCR are listed in Supplementary Table S2.

### RNase H cleavage

To identify all post-transcriptional modifications, LC-purified U2 snRNA (1 pmol) was cleaved in a sequence specific manner with 3 U of RNase H (Takara Bio, Japan) at 42°C for 30 min using a guide DNA oligonucleotide (100 pmol) in 20 μl of 40 mM Tris–HCl (pH 7.7), 4 mM MgCl_2_, 4%(v/v) glycerol and 1 mM DTT. The guide DNA oligonucleotide was designed to hybrid with a region at the 5′ side of 17U in U2 snRNA. Prior to RNase H digestion, the LC-purified U2 snRNA was denatured at 74°C for 3 min, and annealed with guide DNA oligonucleotide by gradually lowering temperature from 74 to 50°C for 12 min. The RNase H digest was directly loaded onto reversed-phase LC columns and purified RNA fragments corresponding to the regions of 1–16 and 17–188 in U2 snRNA. Sequence of the guide DNA oligonucleotide is listed in Supplementary Table S2.

### Direct nanoflow LC-MS and MS/MS (MS2) analysis of RNA fragments

Nucleolytic RNA fragments were analyzed on a direct nanoflow LC-MS system based on the method described by Yamauchi *et al.* with some modifications (45). Briefly, the RNase digests (U1, 5.8S, 18S or 28S) were first concentrated on a trap column (MonoCap C18, 200 μm i.d. × 40 mm; GL Sciences, Tokyo, Japan), eluted with 100 mM triethylammonium acetate (pH 7.0) containing 0.1 mM diammonium phosphate and loaded to a 15 μl sample injection loop. The RNA fragments were separated with a reversed-phase tip column [Develosil C30 UG, 150 μm i.d. × 120 mm (U1, U2, 5.8S) or 240 mm (18S, 28S), 3-μm particle size; Nomura Chemical Co. Ltd, Aichi, Japan] with a 60-min linear gradient from solvent A (10 mM triethylammonium acetate in 10% methanol) to 25% solvent B (10 mM triethylammonium acetate:acetonitrile, 60:40) at a rate of 100 nl/min (U1, U2, 5.8S) or at 200 nl/min (18S, 28S), or with a 120-min linear gradient from solvent A to 25% solvent B (18S, 28S). After the separation, the column was washed with 70% B for 10 min and re-equilibrated with solvent A for the next analysis.

Eluate from a reversed-phase tip column was sprayed online at −1.3 kV with the aid of a spray-assisting device (46) to a Q Exactive Plus Hybrid Quadrupole-Orbitrap Mass Spectrometer (Thermo Fisher Scientific, San Jose, CA, USA) in negative ion mode. Survey full-scan mass spectra (from *m/z* 480 to 1980) were acquired at a mass resolution of 35 000. Higher-energy CID with normalized collision energy of 20 and 50% fragmented precursors. Other settings were as described (45).

### SILNAS-based and peak area-based quantification of post-transcriptional modifications (PTMs)

SILNAS-based quantitation was performed as described by Taoka *et al.* (44). Briefly, for RNase T1 digestion, U1 or U2 snRNA (50 fmol) from cells grown in guanosine with natural isotope distribution was mixed with an equal amount of synthetic internal standard U1 or U2 snRNA with ^13^C-labeled guanosine. The 1:1 RNA mixing was estimated based on the measurement of the absorbance at 260 nm and ensured later by a correction factor obtained experimentally as an average of the quantizations of ∼10 pairs of labeled and non-labeled RNA fragments with the same nucleotide sequences without modifications. The RNase T1 digestion (2 ng/μl) was done in 100 mM triethylammonium acetate buffer (pH 7.0) at 37°C for 60 min. RNase A digestion (0.25 ng/μl) was done with the same procedure with for RNase T1 except using internal standard RNAs synthesized with ^13^C-labeled cytidine and uridine. SILNAS-based quantization of 5.8S, 18S or 28S rRNA were performed as described (44). The stoichiometry of RNA modification at 70A in U1, or 19G, 25G or 34U in U2 snRNA was estimated by Ariadne program as described (44). For the peak area-based quantification of post-transcriptional modification (PTM), the extracted-ion chromatograms with theoretical value (± 5 ppm) of the target oligonucleotides were obtained and the start-end of the peak was manually selected and measured the peak area using Thermo Scientific Xcalibur software (Thermo Fisher Scientific). The RNA sequences of all obtained peaks were confirmed by Ariadne database search and the manual inspection.

### FISH

FISH was carried out based on the method described (37). Briefly, HeLa cells cultured on collagen-coated culture slides (Beckton Dickinson) were fixed with 4% paraformaldehyde in PBS for 20 min, washed twice with PBS and permeabilized for 16 h at 4°C in 75% ethanol. After incubating the slides with 2× SSC containing 15% formamide to rehydrate the cells, hybridization was done with 0.5 ng/μl of fluorescence-labeled (Cy3 or FITC) DNA probe in hybridization solution (15% formamide, 2× SSC, 0.5 mg/ml yeast tRNA, 10% dextran sulfate, 50 mg/ml BSA and 10 mM Ribonucleoside–Vanadyl Complex) for 16 h at 37°C. After hybridization, the cells were sequentially washed twice with 2× SSC and 15% formamide, once with 1× SSC, and once with PBS. After post-fixation with 4% paraformaldehyde in PBS for 10 min, the cells were incubated with the appropriate primary antibody for 2 h at 25°C. After being washed three times with PBS containing 0.05% (w/v) Tween 20 (PBST) for 10 min each, the cells were incubated with a fluorescent-conjugated secondary antibody for 1 h at 25°C. Finally, after being washed three times with PBST for 10 min each, the cells were mounted using VECTASHIELD Mounting Medium with DAPI (Vector Laboratories, Burlingame, CA) and observed with an Axiovert 200 M microscope (Carl Zeiss, Oberkochen, Germany).

### Immunocytochemical staining

Cells grown on collagen-coated culture slides were washed with PBS and fixed with 4% paraformaldehyde in PBS for 10 min at 25°C. After being washed twice with PBST, the cells were permeabilized with PBS containing 0.1% (v/v) Triton X-100 (SIGMA) for 5 min at 25°C and then were washed again with PBST. The cells were blocked with 3% (w/v) non-fat dried skim milk in PBST for 1 h and then were incubated with an appropriate primary antibody for 2 h at 25°C. After three 10-min washes with PBST, the cells were incubated with a fluorescence-conjugated secondary antibody for 1 h at 25°C. Finally, after three additional 10-min washes with PBST, the cells were mounted using VECTASHIELD Mounting Medium with DAPI and examined with an Axiovert 200 M microscope.

### Fractionation of nucleoli

Fractionation of nucleoli was carried out as described (47) with some modifications. Briefly, HeLa cells cultured in a 90-mm culture dish were washed twice with ice-cold PBS, harvested, suspended in 500 μl of Sol I (500 mM sucrose, 3 mM MgCl_2_, 1 mM PMSF) and lysed by sonication using the Bioruptor 200 (Middle setting, five 10-s pulses, with 50-s intervals, 4°C). One-fifth of the solution was used to extract total RNA, and the remaining solution was used to prepare nucleoli. The latter solution was overlaid on 700 μl of Sol II (1 M sucrose, 3 mM MgCl_2_, 1 mM PMSF) and centrifuged at 1700 *g* for 5 min at 4°C, and the supernatant was collected as the cytoplasmic-nucleoplasm fraction and subjected to the following RNA extraction. The remaining pellet was washed once with 700 μl of Sol I and centrifuged at 1700 *g* for 5 min at 4°C; the nucleolar fraction was collected by carefully removing the supernatant, which was subjected to the following RNA extraction. RNA extraction of each fraction was performed with TRIzol reagent. Total RNA was fixed at 4 μg, and equivalent amount of RNA from cytoplasmic-nucleoplasm or nucleolar fraction was used for northern blot analysis.

## RESULTS

### TDP-43 binds a subset of scaRNAs

**Figure 1.**
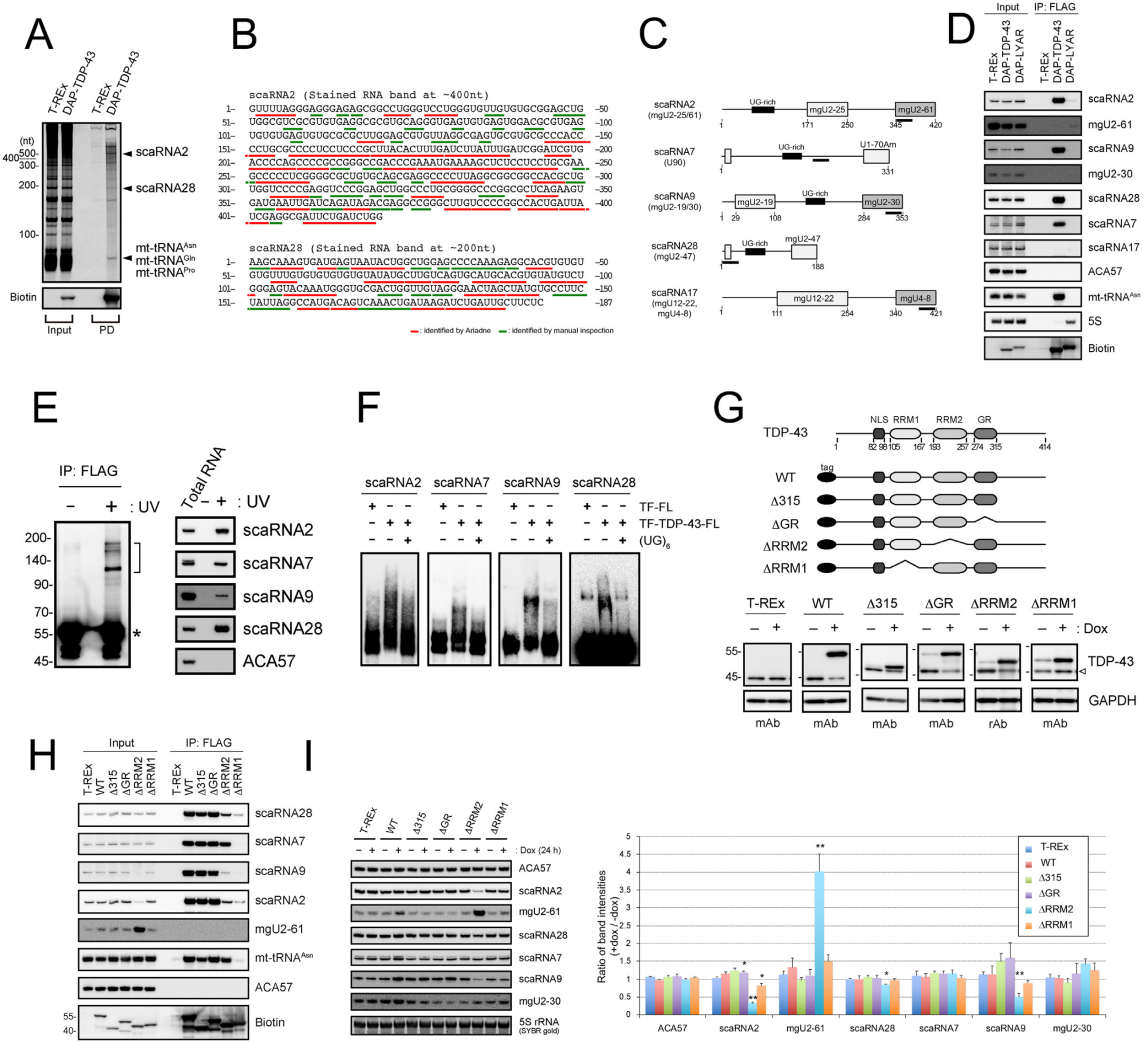
TDP-43 binds C/D scaRNAs that have a UG-rich motif. (**A**) Isolation of RNAs bound to DAP-TDP-43 by two-step affinity purification (pull-down, PD; His-tag and FLAG-tag) from the cell extract of DAP-TDP-43-expressing T-REx 293 cells after a 24-h treatment with doxycycline. DAP-TDP-43 was detected by western blotting using horseradish peroxidase-conjugated streptavidin (streptavidin-HRP; Biotin). scaRNA2 and scaRNA28 were identified by in-gel RNase T1 digestion–LC-MS analysis. Input, total RNA (1 μg/cell line). (**B**) Oligonucleotides identified by LC-MS analysis of the in-gel RNase T1-digested RNA bands corresponding to scaRNA2 and scaRNA28 in Figure 1A are underlined in the sequence of scaRNA2 and scaRNA28. Red lines indicate oligonucleotides identified by Ariadne search, and green lines are by manual inspection. (**C**) Schematic structures of C/D scaRNAs (scaRNA2, 7, 9, 28 and 17). UG-rich motif and the guide domain for post-transcriptional modification are shown in each scaRNA. DNA probes used for northern blot analysis are indicated as black lines under each scaRNA. (**D**) DAP-TDP-43−binding RNAs immunoprecipitated using FLAG antibody (IP: FLAG) were detected by northern blot analysis with DNA probes hybridizing to the RNAs indicated on the right. The parental T-REx 293 cells and DAP-LYAR-expressing T-REx 293 cells were used as controls. DAP-TDP-43 and DAP-LYAR were detected by western blot analysis using streptavidin-HRP (Biotin). Input, total RNA (4 μg/cell line). (**E**) scaRNAs were isolated by the FLAG immunoprecipitation from DAP-TDP-43-expressing T-REx 293 cells with (+) or without (−) UV irradiation, separated by SDS-PGAE (left) and analyzed by northern blot analysis using DNA probes hybridizing to the RNAs indicated on the right (right). Half bracket indicates the position of the excised membrane. RNA-unbound DAP-TDP-43 detected by streptavidin-HRP is indicated by an asterisk. (**F**) Electrophoresis mobility shift assay using each of the biotin-labeled scaRNAs was done with (+) or without (−) recombinant TDP-43 (TF-TDP-43-FL), TF-FL and (UG)_6_ RNA oligonucleotide. (**G**) Schematic structures of DAP-TDP-43 deletion mutants (top). T-REx 293 cells or T-REx 293 cells expressing DAP-TDP-43 (WT) or its deletion mutants (Δ315, ΔGR, ΔRRM2 and ΔRRM1) were treated for 24 h with 100 ng/ml of doxycycline (dox), and the expression levels of DAP-TDP-43, its deletion mutants or endogenous TDP-43 were detected by western blot analysis with anti-TDP-43 antibodies (mouse monoclonal antibody, mAb; rabbit polyclonal antibody, rAb). GAPDH was used as a loading control. Open arrowhead shows endogenous TDP-43. (**H**) RNAs immunoprecipitated with DAP-TDP-43 (WT) and its deletion mutants (Δ315, ΔGR, ΔRRM1, ΔRRM2) using FLAG antibody (IP: FLAG) were detected by northern blot analysis with DNA probes hybridizing to the RNAs indicated on the right. The parental T-REx 293 cells were used as a control. DAP-TDP-43 and its deletion mutants were detected by western blot analysis using streptavidin-HRP (Biotin). Input, total RNA (4 μg/cell line). (**I**) T-REx 293 cells expressing TDP-43 (WT) or domain mutants (Δ315, ΔGR, ΔRRM2 and ΔRRM1) were treated with (+) or without (−) doxycycline for 24 h. Total RNAs were analyzed by northern blotting using DNA probes hybridizing to the RNAs indicated to the right. Ratios of staining band intensities (+dox/−dox) are shown by bar graph for the cells expressing TDP-43 or each of the domain mutants. Mean ± SEM, *n* = 3; *P < 0.05, **P < 0.01, unpaired *t-*test versus T-REx.

DAP-TDP-43-binding RNAs were purified by two-step pull-down method using His6-tag in DAP-tag for the first step and FLAG-tag in DAP-tag for the second step from cell extracts of T-REx 293 cells inducibly expressing DAP-TDP-43 from a chromosomally integrated gene. These RNAs were separated by denaturing urea gel electrophoresis (Figure 1A) and analyzed by a mass spectrometry (MS)-based method in combination with the genome-wide search engine Ariadne after RNase T1 digestion of each of the excised bands that stain RNA (12,40–41). Although we identified a subset of mitochondrial tRNAs (mt-tRNAs) in the stained bands having a size of ∼70 nt as reported (12), we also detected many oligonucleotides in the other two stained bands having ∼400 and 200 nt (Figure 1A). Ariadne mapped those oligonucleotides with the highest scores to regions encoding scaRNA2 and scaRNA28 in the human genome (Figure 1B and Supplementary Table S3). Northern blot analysis using synthetic oligonucleotides complementary to scaRNA2 and scaRNA28 as hybridizing probes confirmed their identification (Figure 1C and D). In addition, ultraviolet cross-linking and immunoprecipitation (UV-CLIP) showed that the interaction was direct without any cofactor required with TDP-43 and scaRNA2 or scaRNA28 (Figure 1E). Given that those two scaRNAs have a UG-rich motif, which is a typical TDP-43-binding motif (6), we examined the binding of TDP-43 via this motif by electrophoresis mobility sift assay using (UG)_6_ oligonucleotide as an RNA antagonist and demonstrated that (UG)_6_ oligonucleotide interrupted the binding of those scaRNAs to TDP-43 (Figure 1F), suggesting that UG-rich motifs in scaRNA2 and scaRNA28 are responsible for TDP-43 binding.

Among 28 known human scaRNAs (scaRNA1–28), two scaRNAs—scaRNA7 (U90) and scaRNA9—are predicted to have UG-rich motifs (Supplementary Table S4) (29,32,33,35,48,49). Indeed, those two scaRNAs interacted with TDP-43 (Figure 1C and D), whereas TDP-43 bound neither scaRNA17 (C/D scaRNA), ACA57 (H/ACA scaRNA) nor 5S rRNA, none of which have the UG-rich motifs (Figure 1D and Supplementary Table S4). UV-CLIP and electrophoresis mobility sift assay using (UG)_6_ oligonucleotide confirmed that scaRNA7 and scaRNA9 also directly bound TDP-43 via UG-rich motifs (Figure 1E and F). As all four of the scaRNAs that have UG-rich motifs belong to the family of C/D scaRNAs (Supplementary Table S4), we suggest that TDP-43 binds specifically to a subset of C/D scaRNAs containing the UG-rich motif.

To locate the region bound to the C/D scaRNAs in the TDP-43 molecule, in addition to the DAP-TDP-43-expressing cell line, we established T-REx 293 cell lines that inducibly expressed each of four domain-deletion mutants having nuclear localization signals (Δ315, ΔGR, ΔRRM2 and ΔRRM1) (Figure 1G). We pulled down each of those mutants with FLAG-tag individually as affinity bait and showed that RRM1 is essential for binding to each of the four C/D scaRNAs, whereas the contribution of RRM2 varied among these C/D scaRNAs (Figure 1H). RRM2 was essential for the binding of TDP-43 to scaRNA9 and contributed to some extent to the binding to scaRNA2 or scaRNA28, but RRM2 had almost no contribution to the binding of scaRNA7. In addition, we noted that the expression of ΔRRM2 was associated with a reduction in scaRNA2 and scaRNA9 and an increase in mgU2-61, a RNA fragment generated by the processing of scaRNA2 (Figure 1H and I) (50,51).

### TDP-43 regulates site-specific 2′-*O*-methylation of U1 and U2 snRNAs

**Figure 2.**
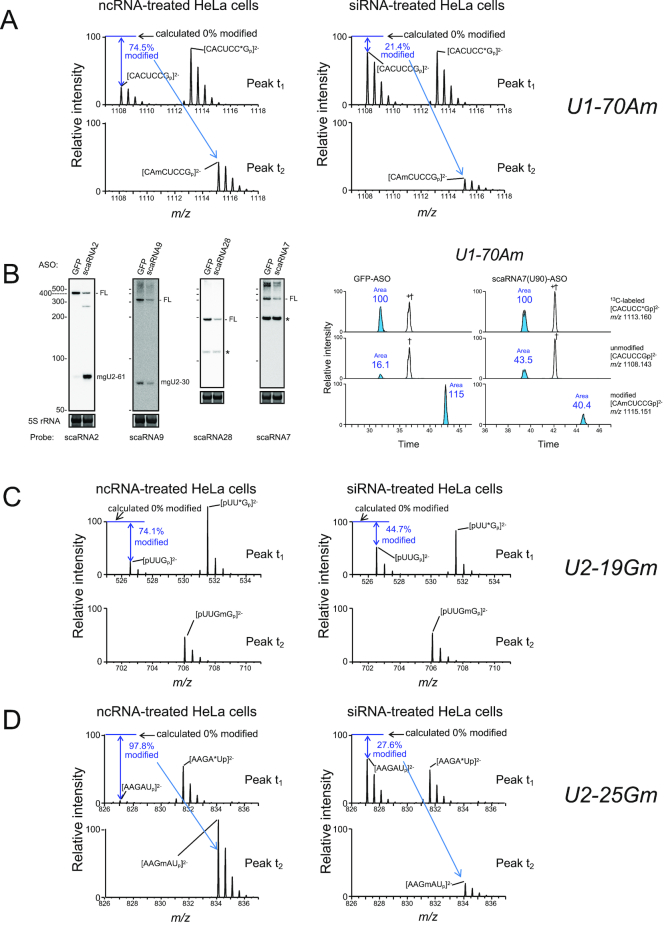
TDP-43 regulates site-specific 2′-*O*-methylations of U1 and U2 snRNAs. (**A**) SILNAS analysis of the level of 70Am in U1 snRNA. MS spectra of [CACUCCGp]^2−^, [CACUCC*Gp]^2−^ and [CAmCUCCGp]^2−^ ions were obtained by SILNAS analysis of U1 snRNA prepared from HeLa cells treated for 96 h with ncRNA (left, control) or siRNA (right, TDP-43 knockdown). MS spectra of the peaks eluted at retention times of t_1_ (upper) and t_2_ (lower) in the chromatograms of Supplementary Figure S1A are given. From the signal intensity of MS spectra of the light [CACUCCGp]^2−^ ion relative to that of the heavy [CACUCC*Gp]^2−^ ion (as an internal control), the extent of 2′-*O*-methylation of the ion generated from U1 snRNA in ncRNA- and siRNA-treated cells was estimated as 74.5% and 21.4%, respectively. The corresponding methylated ion was detected in the MS spectrum of the peak t_2_. (**B**) HeLa cells were treated for 96 h with an antisense oligonucleotide (ASO) against scaRNA2, 7, 9, 28 or GFP mRNA (control), and the knockdown efficiency of each scaRNA was examined by northern blot analysis with DNA probes complementary to the RNAs indicated under each figure (left). FL indicates a band corresponding to the full-length wild-type scaRNA. Non-specific bands are shown as asterisks. 5S rRNA stained with SYBR Gold is shown as the loading control. Extracted ion monitoring of RNase T1-digested fragments of U1 containing 2′-*O*-methylation is shown for quantitative analysis upon the knockdown of scaRNA7 (right). (**C** and **D**) MS spectra of [pUUGp]^2−^, [pUU*Gp]^2−^ and [pUUGmGp]^2−^ ions for 19Gm (C) and [AAGAUp]^2−^, [AAGA*Up]^2−^ and [AAGmAUp]^2−^ ions for 25Gm (D) were obtained by SILNAS analysis of U2 snRNA prepared from HeLa cells treated for 96 h with ncRNA (left) or siRNA (right) for TDP-43 knockdown. MS spectra of the peaks eluted at retention times of t_1_ (upper) and t_2_ (lower) in the chromatograms of Supplementary Figure S2B are shown. From the signal intensity of MS spectra of the light and heavy [pUUGp]^2−^ (C) or [AAGAUp]^2−^ (D) ion, the extent of 2′-*O*-methylation in ncRNA- and siRNA-treated cells was estimated.

Given that C/D scaRNAs are expected to guide site-specific 2′-*O*-methylation of snRNAs (Supplementary Table S4)—i.e. scaRNA7 guides the 2′-*O*-methylation presumably at 70A in U1 snRNA; scaRNA2 at 11G, 25G and 61C in U2 snRNA; scaRNA9 at 19G and 30A in U2 snRNA; and scaRNA28 at 47U in U2 snRNA—we considered whether TDP-43 binding has a possible role in the methylation reactions of U1 or U2 snRNA via these C/D scaRNAs. We used a quantitative liquid chromatography (LC)-MS-based method, SILNAS (52), to analyze these methylation reactions under the condition of TDP-43 knockdown with small interfering RNAs (siRNAs) for 96 h in HeLa and 293T cells. Given that U1 or U2 snRNA has long half-life of 2 to 4 days (53), we treated those cells for 96 h with siRNA to ensure enough effects on the modification of U1 or U2 snRNA upon TDP-43 knockdown. For this purpose, we incorporated an *in vitro*-transcribed, heavy isotope-labeled reference U1 or U2 snRNA into a corresponding U snRNA sample solution, digested the mixture with RNase T1 or RNase A and detected the post-transcriptionally modified oligonucleotides quantitatively based on shifts in retention time and the MS signals in subsequent LC-MS analyses. In this analysis, for U1 snRNA, for example, RNase T1 is expected to produce three fragments for the 70A methylation site if the site is partially methylated: an unmodified CACUCCG_69−75_ and a modified CAmCUCCG_69−75_, both of which originated from the sample U1 snRNA, and an unmodified CACUCC*G_69−75_ (*G, ^13^C_10_-guanosine) from the heavy isotope-labeled reference U1 snRNA. In fact, we detected the MS spectra corresponding to those three fragments from U1 snRNA prepared from HeLa cells treated with the non-specific control RNA (ncRNA) (Figure 2A, left); the two unmodified fragments were eluted at t_1_, and the modified one was eluted at t_2_ during LC separation (Supplementary Figure S1A). Both the calculations based on the MS intensities of those fragments (Figure 2A and Supplementary Table S5), and those based on the peak areas of chromatograms (Supplementary Figure S1A) were consistent in that ∼75% of the U1 snRNA molecules were methylated at 70A in ncRNA-treated HeLa or 293T cells (Table 1; Supplementary Tables S5 and S6). In contrast, ∼25% were methylated in siRNA-treated cells (Figure 2A, right and Table 1;Supplementary Figure S1A, and Supplementary Tables S5 and S6). Coincidently, RNase T1 produced ACCCCUG_85−91_ and ACCCCU*G_85−91_ from U1 snRNA, which had the same *m/z* values as CACUCCG_69−75_ and CACUCC*G_69−75_, respectively, eluted at t_3_ (Supplementary Figure S1A, shown as † and +†, respectively; the identification was done by subsequent MS/MS analysis.). The latter two fragments were not modified and their MS intensities did not change upon knockdown of TDP-43 (Supplementary Figure S1A), which also did not affect the 2′-*O*-methylation at 1A and 2U of U1 snRNA as examined as a negative control (Table 1 and Supplementary Table S6). The knockdown of scaRNA7 using an antisense oligonucleotide (ASO) against scaRNA7 reduced the 2′-*O*-methylation at 70A (Table 1 and Figure 2B), suggesting that TDP-43 regulates selectively the 2′-*O*-methylation at 70A via its action on scaRNA7.

**Table 1.** Quantification of site-specific methylation

Modified nucleotide	Modified oligonucleotide	HeLa (% modification)^a^			HeLa (% modification)^b^		Guide scaRNA (predicted)	Quantitative methods
		ncRNA	siRNA-1	siRNA-2	GFP-ASO	scaRNA-ASO		
**U1 snRNA**								
Cap (1Am, 2Um)	TMG-AmUmACYYACCUG	100 ± 0	100 ± 0	100 ± 0			scaRNA16, 18	Peak area
70Am	CAmCUCCG	79.3 ± 3.9	24.2 ± 2.3**	23.4 ± 0.7**	83.9	59.6	scaRNA7	SILNAS
70Am	CAmCUCCG	78.1 ± 5.6	20.3 ± 2.4**	19.2 ± 3.1**	87.7	48.2	scaRNA7	Peak area
**U2 snRNA**								
Cap (1Am, 2Um)	TMG-AmUmC	99.3 ± 0.0	99.1 ± 0.1	99.2 ± 0.1			–	Peak area
11Gm	GmGmC + GmGC	95.3 ± 1.6	92.6 ± 2.7	96.1 ± 0.6	96.9	87.9	scaRNA2	Peak area
12Gm	GmGmC + GGmC	96.2 ± 1.3	93.8 ± 2.3	96.4 ± 0.5			unknown	Peak area
19Gm	UUGmG	76.5 ± 1.4	47.7 ± 2.4*	53.9 ± 4.4*	85.1	57.8	scaRNA9	SILNAS
25Gm	AAGmAU	98.0 ± 1.0	33.9 ± 5.4**	33.1 ± 4.4**	98.3	65.1	scaRNA2	SILNAS
25Gm	AAGmAU	98.7 ± 0.7	35.5 ± 7.4**	29.4 ± 3.5**	98.5	66.3	scaRNA2	Peak area
30Am	Am6AmGU + AAmGU	94.8 ± 1.6	90.9 ± 0.9	93.8 ± 0.6	94.9	86.3	scaRNA9	Peak area
34Ψ	ΨAG	84.0 ± 6.0	79.5 ± 5.4	86.0 ± 6.0			scaRNA8	SILNAS
34Ψ	ΨAG	84.5 ± 6.9	82.5 ± 6.0	82.8 ± 7.8			scaRNA8	Peak area
40Cm	YAYCmYG	95.1 ± 2.5	73.7 ± 2.0**	63.6 ± 0.7**			unknown	Peak area
47Um	YYCUUmAUCAG	95.6 ± 0.8	27.1 ± 1.9**	24.5 ± 1.7**	97.0	54.2	scaRNA28	Peak area
61Cm	UYUAAYAUCmUG	57.2 ± 8.9	20.6 ± 1.4*	16.8 ± 0.6*	65.6	45.3	scaRNA2	Peak area

m; methyl group, Y; U or pseudouridine (Ψ), TMG; 2,2,7-trimethylguanosine (m3G).

a: Values are obtained as mean ± SEM (*n* = 3). *P < 0.05; **P < 0.01 (Tukey’s test; versus ncRNA). b: Values are given as a single analysis.

*TDP-43* mRNA levels (per *GAPDH* mRNA); ncRNA (1.000 ± 0): siRNA-1 (0.248 ± 0.025): siRNA-2 (0.244 ± 0.013).

In the case of analysis U2-cap, 11Gm, 12Gm and 19Gm; ncRNA (1.000 ± 0), siRNA-1 (0.274 ± 0.025), siRNA-2 (0.288 ± 0.022).

In addition, the knockdown of TDP-43 reduced 2′-*O*-methylation at 19G, 25G, 47U and 61C in U2 snRNA in HeLa and 293T cells, whereas it did not affect the other analyzed 2′-*O*-methylation sites of U2 snRNA (Figure 2C and D, Table 1;Supplementary Figure S1B, Supplementary Tables S5 and S6). The reductions coincided with those caused by the knockdown of scaRNA2, scaRNA9 or scaRNA28 using the corresponding ASOs (Figure 2B and Table 1; Supplementary Figure S1C), suggesting that TDP-43 also regulates 2′-*O*-methylation at these positions via its action on scaRNA2, scaRNA9 and scaRNA28. In contrast, although scaRNA2 and scaRNA9 were also expected to guide 2′-*O*-methylation at 11G and 30A in U2 snRNA, respectively, knockdown of TDP-43, scaRNA2 or scaRNA9 affected methylation at this position only marginally (Table 1), suggesting that TDP-43, scaRNA2 or scaRNA9 is not involved in these methylations. For scaRNA2, the result is consistent with the fact that its proposed target selection for G11 modification and the following ‘D’ box’ sequences are not conserved throughout species. Because scaRNA2 and scaRNA9 seem to contribute only a little, if at all, to the 2′-*O*-methylation at the respective 11G and 30A in U2 snRNA, other scaRNAs may contribute to the 2′-*O*-methylation of those nucleotides. In addition, it should be noted that we observed an unexpected but substantial decrease (20−30%) in the 2′-*O*-methylation at 40C in U2 snRNA in HeLa cells (Table 1 and Supplementary Figure S1B). In overall, the present analyses show that modifications in U1 or U2 snRNAs range from 99% to as low as 57%. To our knowledge, this is the first time that the variability in snRNA modification is shown at these precisions.

### TDP-43 suppresses the processing of scaRNA2

**Figure 3.**
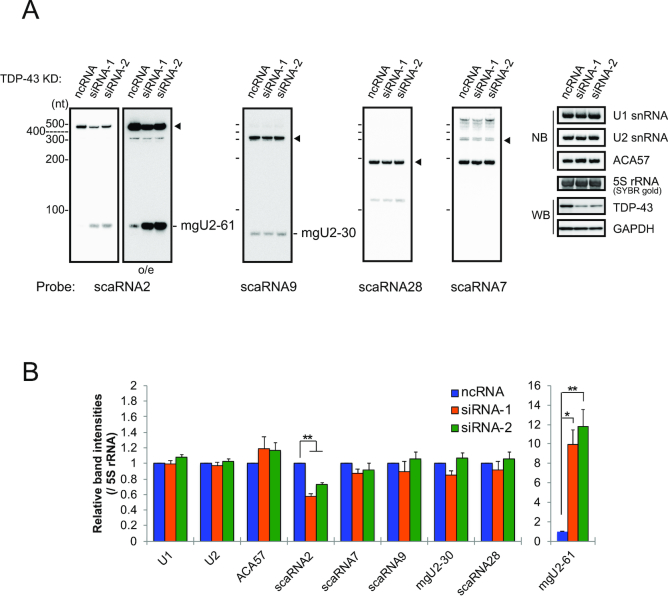
TDP-43 stabilizes scaRNA2 and suppresses the formation of mgU2-61. (**A**) RNAs prepared from 293T cells treated with siRNA-1, siRNA-2 or ncRNA (control) for 96 h for TDP-43 knockdown were detected by northern blotting with DNA probes shown in Figure 1C. The levels of TDP-43 were examined by western blot analysis with anti-TDP-43. Closed arrowhead indicates full-length scaRNAs. (**B**) The graph shows the relative band intensities of RNAs that were normalized with that of 5S rRNA detected by SYBR gold staining. Mean ± SEM, *n* = 3–4; *P < 0.05, **P < 0.01 by Tukey’s test versus ncRNA.

To examine how TDP-43 regulates the function of C/D scaRNA as guide RNAs in the 2′-*O*-methylation reaction of U1 or U2 snRNA, we first considered the possibility that TDP-43 stabilizes the bound scaRNAs. Knockdown of TDP-43 reduced full-length scaRNA2 but increased a scaRNA2 fragment of ∼75 nt corresponding to mgU2-61 in 293T and HeLa cells (Figure 3 and Supplementary Figure S2), suggesting that TDP-43 stabilizes scaRNA2 and suppresses the formation of mgU2-61. This effect was very similar to that caused by the expression of ΔRRM2 (Figure 1H and I). As expected from the report that the RRM1 domain is responsible for autoregulation of *TDP-43* mRNA expression (7), increased expression of ΔRRM2, Δ315 and ΔGR reduced endogenous TDP-43 (Figure 1G). Therefore, the expression of those domain mutants should have an effect on the production of mgU2-61 similar to that of TDP-43 depletion; however, the expression of Δ315 and ΔGR did not (Figure 1H and I). Given that Δ315 and ΔGR both have RRM1 and RRM2 but ΔRRM2 has only RRM1, and RRM1 is essential to bind scaRNA2 (Figure 1H), we assume that both RRM1 and RRM2 are required for protecting scaRNA2 from ribonucleolytic degradation and suppressing the formation of mgU2-61. In contrast, the knockdown did not have any substantial effects on the other C/D scaRNAs, although scaRNA9 and scaRNA7 were observed as multiple molecular species (Figure 3 and Supplementary Figure S2).

### TDP-43 regulates the CB localization of a subset of C/D scaRNAs

**Figure 4.**
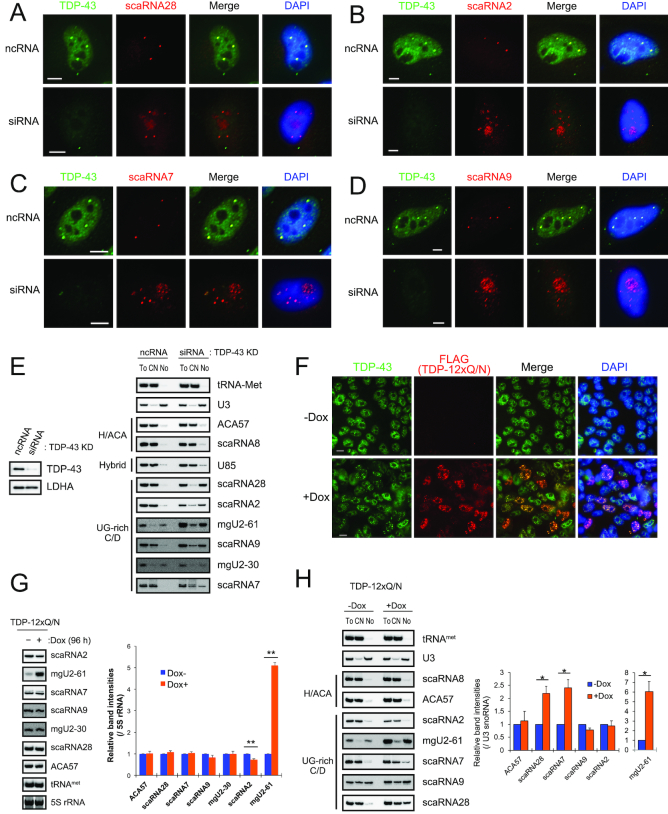
TDP-43 maintains CB localization of UG-rich motif-bearing C/D scaRNAs. (**A***–***D**) FISH analyses for TDP-43 and scaRNA28 (A), scaRNA2 (B), scaRNA7 (C) and scaRNA9 (D) with and without TDP-43 knockdown in HeLa cells. After a 48-h incubation with ncRNA or TDP-43 siRNA, each scaRNA was ectopically expressed for an additional 48 h and detected with Cy3-labeled DNA probes (red). TDP-43 was detected with rabbit polyclonal anti-TDP-43 and FITC-conjugated anti-rabbit IgG (green). Bars indicate 5 μm. FITC and Cy3 signals were merged (Merge); DAPI for DNA staining was overlaid with the merged image. (**E**) HeLa cells treated for 96 h with ncRNA or TDP-43 siRNA were separated into the cytoplasmic-nucleoplasmic fraction (CN) and the nucleolar fraction (No). RNAs extracted from the CN, No or total cell (To) were detected by northern blot analysis with DNA probes complementary to the RNAs indicated on the right. The extent of TDP-43 knockdown was determined by western blot analysis with antibody against TDP-43 or LDHA (control). (**F**) Immunocytochemical analysis for TDP-43 and FLAG-TDP-12xQ/N in FLAG-TDP-12xQ/N-expressing T-Rex 293 cells after 96-h treatment of doxycycline. TDP-43 or FLAG-TDP-12xQ/N was detected with rabbit polyclonal anti-TDP-43 and anti-rabbit IgG FITC-conjugated (green), or mouse monoclonal anti-FLAG IgG and anti-mouse IgG Cy3-conjugated antibody (red). Bars indicate 5 μm. FITC and Cy3 signals were merged (Merge); DAPI for DNA staining was overlaid with the merged image. (**G**) Northern blot analysis of scaRNAs in FLAG-TDP-12xQ/N in FLAG-TDP-12xQ/N-expressing T-Rex 293 cells with (+) or without (-) doxycycline (Dox) treatment. RNAs were detected with DNA probes complementary to the RNAs indicated on the right. The graph shows the band intensities of RNAs for (+) relative to those for (-) detected by northern blot analysis. The band intensity of each of RNAs was normalized with that of 5S rRNA detected with SYBR gold staining. Mean ± SEM, *n* = 3–4; **P < 0.01, paired *t-*test. (**H**) FLAG-TDP-12xQ/N-expressing T-REx 293 cells with (+Dox) or without (-Dox) Dox treatment were separated into the CN and No. RNAs extracted from CN, No or To were detected by northern blot analysis with DNA probes complementary to the RNAs indicated on the right. The graph shows the band intensities of scaRNAs for +Dox relative to those for -Dox in No fractions. The values were normalized with that of U3 (snoRNA). Mean ± SEM, *n* = 3–4; *P < 0.05, paired *t-*test.

Given that CBs represent a nuclear organelle in which scaRNAs localize and perform site-specific post-transcriptional modifications of snRNAs (28,29,32,33), we next considered the possibility that TDP-43 affected localization of these C/D scaRNAs to the CBs. We carried out fluorescence *in situ* hybridization (FISH) analyses of cells that ectopically expressed one of the four C/D scaRNAs. Knockdown of TDP-43 in these cells led to re-localization of all four C/D scaRNAs from the CBs mainly to the nucleolus (Figure 4A–D and Supplementary Figure S3A–D). The knockdown also reduced the number of CBs per cell (Supplementary Figure S3E) as reported (10,18). In addition, the increased nucleolar localization of the four endogenous scaRNAs but not of H/ACA scaRNAs (ACA57, scaRNA8 and U85) was confirmed by northern blot analysis following the nucleolar fractionation of the TDP-43-knock downed HeLa cells (Figure 4E); thus, TDP-43 has a novel role in maintaining the localization of these C/D scaRNAs in CBs. Collectively, our data suggest that TDP-43 regulates site-specific 2′-*O*-methylation of U1 and U2 snRNAs via its ability to maintain localization of scaRNA7, scaRNA2, scaRNA9 and scaRNA28 in CBs.

TDP-43 aggregation is a hallmark of TDP-43 proteinopathies observed in most of the patients of ALS and Frontotemporal Lobar degeneration (FTLD) and believed to cause ‘loss of function’ of TDP-43 via sequestering from its normal localization sites. Therefore, we expected that TDP-43-aggregation and the following TDP-43 loss of function might cause the de-localization of the C/D scaRNAs in CBs observed by the depletion of TDP-43. To examine this, we used a cell model causing TDP-43 aggregation by expressing TDP-12xQ/N that has tandem repeats of the prion-like Q/N-rich region of TDP-43 fused to additional TDP-43 protein sequences to trigger the formation of the aggregate of endogenous TDP-43 (54). In this cell model, TDP-12xQ/N formed a number of foci in the nucleus as reported (55) (Figure 4F) and caused the decrease of scaRNA2 and the increase of mgU2-61 (Figure 4G). In addition, the expression of TDP-12xQ/N increased the amounts of scaRNA7 and scaRNA28 in the nucleolar fractions (Figure 4H) and caused the nucleolar localization of scaRNA7 and scaRNA28 (Supplementary Figure S3F), suggesting that the expression of TDP-12xQ/N causes ‘loss of function’ of TDP-43 in terms of its ability to localize a subset of C/D scaRNAs in CBs.

Given that TDP-43 regulates the formation and nucleolar accumulation of mgU2-61 (from scaRNA2) (Figure 4E and H), which is predicted to obstruct the role of HBII-82B (which guides 2′-*O*-methylation at 3923G in 28S ribosomal RNA) via complementary hybridization (51), we analyzed the 2′-*O*-methylation at 3923G in 28S rRNA by quantitative LC-MS after TDP-43 knockdown; however, the knockdown had no effect on the methylation (Supplementary Table S7). In addition, as the other TDP-43-binding C/D scaRNAs contain sequences that are predicted to inhibit or guide 2′-*O*-methylation or pseudouridylation at specific sites in ribosomal 28S, 18S and 5.8S RNAs (Supplementary Table S7) (51,56), we examined each of those modifications by quantitative LC-MS but did not observe any changes in the modifications at any of these sites (Supplementary Table S7).

### WDR79 is not required for the CB localization of a subset of C/D scaRNAs

**Figure 5.**
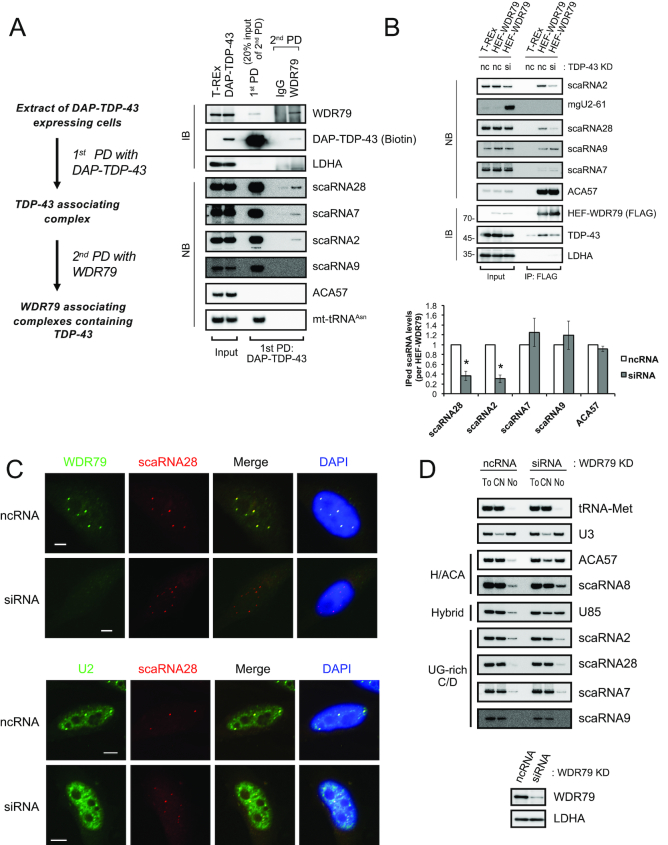
WDR79 is not involved in CB localization of UG-rich motif-bearing C/D scaRNAs. (**A**) Isolation of WDR79−TDP-43 complex by two-step pull-down (PD) method. DAP-TDP-43 complexes were first pulled down (1st-PD) with anti-FLAG from DAP-TDP-43-expressing T-REx 293 cells (induced with doxycycline for 24 h) and then pulled down (2nd-PD) with anti-WDR79 (protocol is shown at left). Protein components were detected by western blot analysis (IB) with anti-WDR79 or streptavidin-HRP (biotin). RNAs were detected by northern blot analysis (NB) with DNA probes complementary to the RNAs indicated on the right. Input consisted of 10 μg of cell lysate or 4 μg of RNA extracted from cell lysates. (**B**) HEF-WDR79-expressing T-REx 293 cells were treated with ncRNA (nc) or TDP-43 siRNA (si) for 72 h and further treated with Dox for 24 h. RNAs and proteins associated with HEF-WDR79 were immunoprecipitated with anti-FLAG antibody (IP: FLAG). Protein components were detected by western blot analysis (IB), and RNAs were by northern blot analysis (NB) with DNA probes complementary to the RNAs indicated on the right. Input, 10 μg of cell lysate or 4 μg of RNA extracted from cell lysates. The graph shows the band intensities of the scaRNAs immunoprecipitated from siRNA-treated cells relative to those from ncRNA-treated cells. The values are normalized with those of the immunoprecipitated (IPed) HEF-WDR79. Mean ± SEM, *n* = 3; *P < 0.05, paired *t-*test. (**C**) Immunocytochemical (WDR79) and FISH (scaRNA28) analysis of HeLa cells treated with ncRNA or siRNA for WDR79 knockdown (upper). FISH analyses of U2 snRNA and scaRNA28 are also given for the HeLa cells treated with ncRNA or siRNA for 96 h for the WDR79 knockdown (lower). In those analyses, scaRNA28 was expressed ectopically for an additional 48 h after the WDR79 knockdown and detected with Cy3-labeled scaRNA28 DNA probe (red). U2 snRNA was detected with a FITC-labeled U2 snRNA DNA probe (green). WDR79 was detected with rabbit polyclonal anti-WDR79 and FITC-conjugated anti-rabbit IgG (green). Bars indicate 5 μm. FITC and Cy3 signals were merged (Merge); DAPI for DNA staining was overlaid with the merged image. (**D**) HeLa cells treated for 96 h with ncRNA or WDR79 siRNA were separated into the cytoplasmic/nucleoplasmic fraction (CN) and the nucleolar fraction (No). RNAs extracted from the CN, No or total cell (To) were detected by northern blot analysis with DNA probes complementary to the RNAs indicated on the right. The extent of the WDR79 knockdown was determined by western blot analysis with antibody against WDR79 or LDHA (control).

Given that WD40-repeat protein 79 (WDR79) drives the CB-specific localization of scaRNA7 and scaRNA28 (mgU2-47) via binding to the G.U/U.G wobble base-pairs in their UG-rich motifs (36), we determined whether TDP-43 competes, assists or works independently of WDR79 with respect to the binding to the UG-rich motifs of these C/D scaRNAs. Initially, we postulated that TDP-43 might enhance the binding between WDR79 and C/D scaRNAs. To address this, we examined first the binding between TDP-43 and WDR79 by pull-down assay using DAP-TDP-43 and its domain mutants as affinity bait. TDP-43 interacted with WDR79 independently of RNA, probably via its RRM1, GR and aa 315-414 domains (Supplementary Figure S4A and S4B). However, reciprocal pull-down using endogenous WDR79 or HEF (HA, TEV cleavage site and FLAG)-WDR79 as affinity bait recovered only a small population of TDP-43 molecules in the cell (Supplementary Figure S4C and S4D). In contrast, WDR79 bound a H/ACA scaRNA (ACA57) several-fold more efficiently than the C/D scaRNAs (Supplementary Figure S4D), as reported (32,33,35). Serial two-step pull-down analysis using DAP-TDP-43 as the first affinity bait and endogenous WDR79 as the second (Figure 5A, see protocol at left) also recovered only a small portion of the TDP-43 complex containing scaRNA28, 7 and/or 2, but not containing a H/ACA scaRNA (ACA57) (Figure 5A right). We did not detect scaRNA9 in this complex (Figure 5A). Pull-down analysis with HEF-WDR79 before and after the TDP-43 knockdown showed that the WDR79 binding to scaRNA2 and scaRNA28—but not to scaRNA9 and scaRNA7—was dependent on TDP-43 (Figure 5B), suggesting that TDP-43 enhances, at least in part, the binding of a small population of scaRNA2 or scaRNA28 to WDR79. Collectively, in turn, these data suggest that the remaining population of scaRNA2 and scaRNA28 and the most of the population of scaRNA9 and scaRNA7 bind to TDP-43 independently of WDR79.

We, therefore, examined further the possibility that TDP-43 and WDR79 contributions to CB localization differ between C/D scaRNAs and H/ACA scaRNAs. Indeed, FISH analyses showed that WDR79 knockdown changed coilin localization into the nucleolus as reported (57) (Supplementary Figure S4E), whereas it did not affect the localization of scaRNA28 to CB-like structures in which U2 snRNA was co-localized (Figure 5C). The WDR79 knockdown also did not affect the localizations of the other three scaRNAs (scaRNAs 2, 7 and 9) to CB-like structures (Supplementary Figure S4F). In addition, WDR79 knockdown did not increase the amounts of these four C/D scaRNAs in the nucleolar fraction, whereas ACA57, scaRNA8 and U85 scaRNAs were increased in the nucleolar fraction as reported (35) (Figure 5D). Collectively, these data and those obtained for the knockdown of TDP-43 (Figure 4A–E) strongly suggest that TDP-43 is a major contributor to the CB localization of the UG-rich motif-bearing C/D scaRNAs.

## DISCUSSION

**Figure 6.**
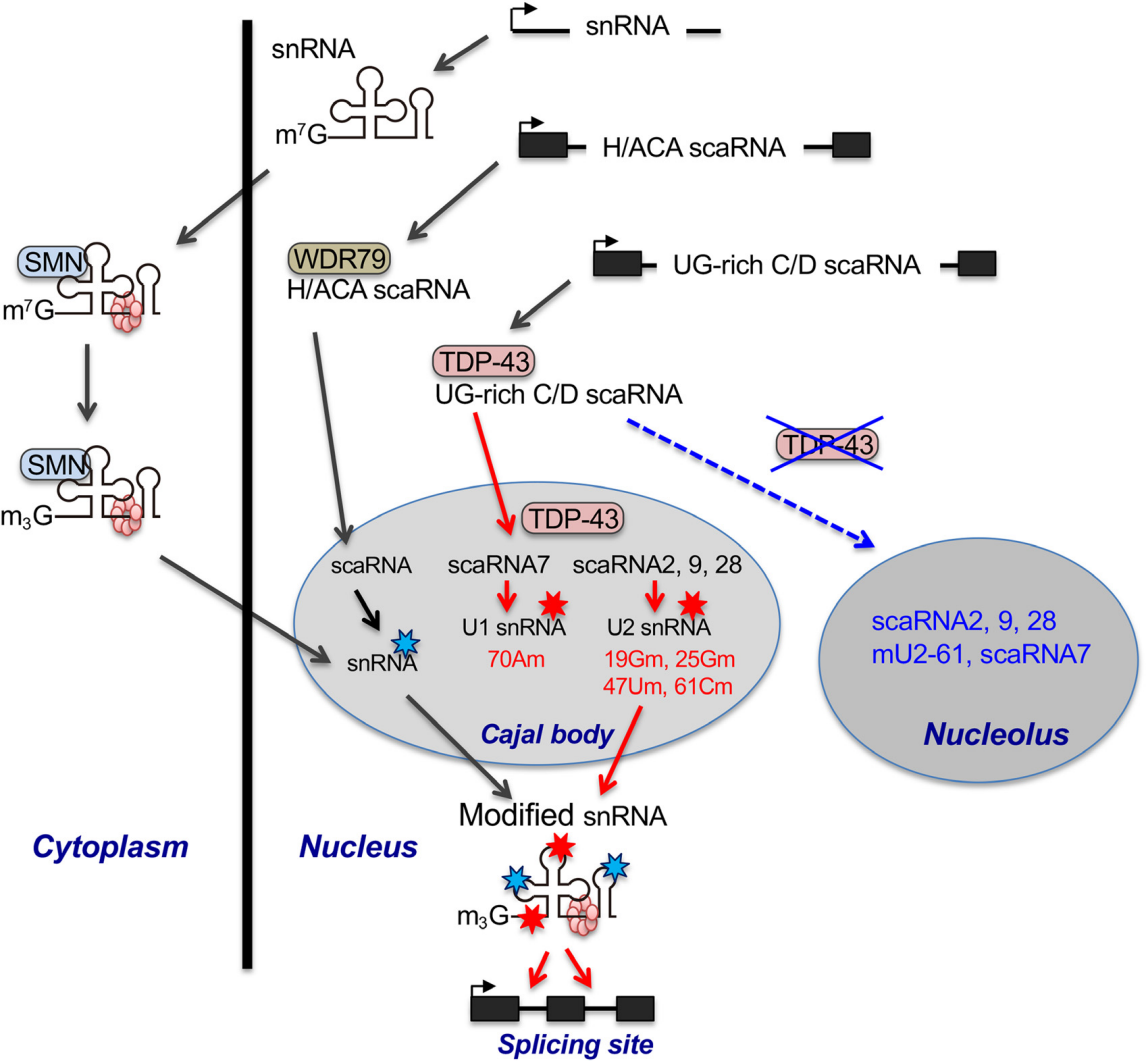
Proposed roles of TDP-43 in post-transcriptional modifications of U1 and U2 snRNAs via the trafficking of UG-rich motif-bearing C/D scaRNAs to CBs. WDR79 transports H/ACA scaRNAs to CBs via binding to the CAB box of H/ACA scaRNAs that guide site-specific pseudouridylation of snRNA. In contrast, TDP-43 assists with the CB localization of UG-rich motif-bearing C/D scaRNAs (UG-rich C/D scaRNAs), including scaRNA7, scaRNA2, scaRNA9 and scaRNA28. In CBs, scaRNA7 guides the 2′-*O*-methylation of 70A in U1 snRNA, whereas scaRNAs 2, 9 and 28 guide the 2′-*O*-methylation of 25G/61C, 19G and 47U in U2 snRNA, respectively. The modified snRNAs (pseudouridylation and 2′-*O*-methylation are indicated by sky blue stars and red stars, respectively) are incorporated into the spliceosome as U snRNPs to perform the splicing reaction. Depletion of TDP-43 causes de-localization of the CB-localized UG-rich C/D scaRNAs into the nucleolus, resulting in the reduction of the site-specific 2′-*O*-methylations of U1 and U2 snRNAs.

It has been reported that WDR79 regulates the localization of H/ACA scaRNAs to CBs and participates in the regulation of CB localization of C/D scaRNAs. Here, we demonstrated that WDR79 and TDP-43 are involved in the CB localization of a small subpopulation of scaRNA2 and scaRNA28, whereas TDP-43, but not WDR79, drives the CB localization of the remaining large populations of the UG-rich motif-bearing C/D scaRNAs. This explains why WDR79 is associated with C/D scaRNAs several-fold less-efficiently than with H/ACA scaRNAs (35). Thus, we suggest that TDP-43 and WDR79 have separate roles in determining CB localization of these C/D and H/ACA scaRNAs (Figure 6). It is not yet clear whether the CB localization of the C/D scaRNAs is driven by TDP-43 alone or by TDP-43 with the assistance of a CB localizing factor(s) other than WDR79 that is yet unknown. In either case, TDP-43 regulates the site-specific 2′-*O*-methylations of U1 and U2 snRNAs through its ability to maintain CB localization of the UG-rich motif-bearing C/D scaRNAs scaRNA2, scaRNA7, scaRNA9 and scaRNA28 (Figure 6).

Although CBs are generally believed to have roles as platforms for the assembly and RNA modification of snRNPs, PTMs can take place without CBs in cell lines, such as those that are originated from coilin-knockout (KO) mouse or those from coilin-KO Drosophila (21,24,42). In the former case, U2 snRNA is co-localized with box C/D type U85 (scaRNA10) in CB-like structure instead of CBs and subjected to PTMs (21,42). We suppose that snRNA and guide RNA meet together in CBs or CB-like structure is a requirement for receiving PTMs of snRNA. Thus, we suggest that TDP-43 has roles in localizing UG-rich-bearing scaRNAs (scaRNA2, 7, 9 and 28) in the CBs or CB-like structures, or in preventing the scaRNAs from localizing into the nucleolus.

**Figure 7.**
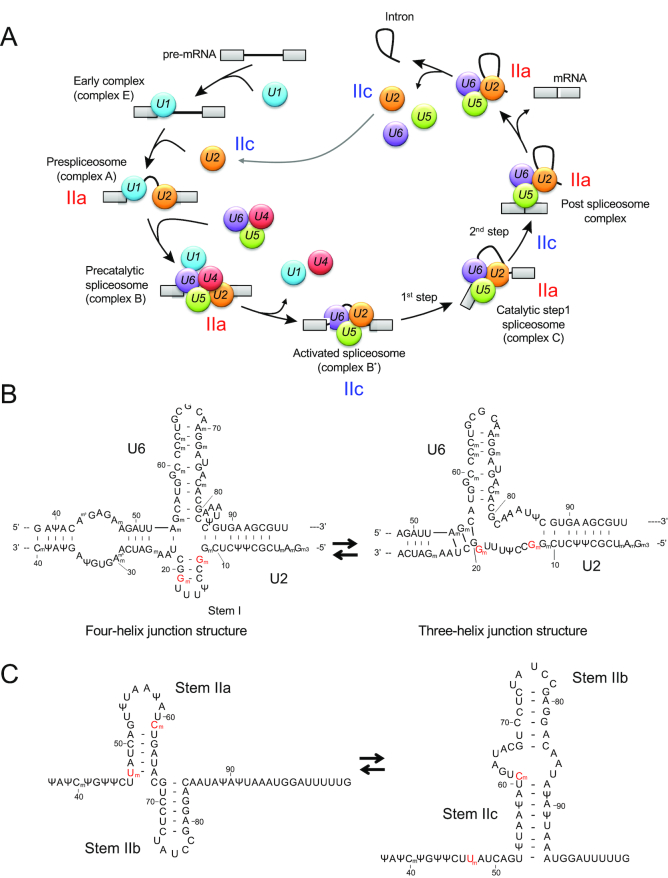
Schematic diagram of pre-mRNA splicing. (**A**) Schematic model of pre-mRNA splicing. U1, U1 snRNP; U2, U2 snRNP; U4, U4 snRNP; U5, U5 snRNP; and U6, U6 snRNP. IIa, stem IIa form of U2 snRNA; IIc, stem IIc form of human U2 snRNA (see Figure 7C). First step; first esterification reaction, second step; second esterification reaction. (**B**) Proposed four-helix (left) and three-helix (right) junction structures of human U2 snRNA in the U2−U6 snRNA complex. The structures are drawn based on yeast U2 snRNA models (65,74). 2′-O-methylated 12G and 19G are shown in red letters. (**C**) Structures of stem IIa and stem IIc forms of human U2 snRNA. The structures are drawn based on the proposed structures of yeast U2 snRNA (76). 2′-O-methylated 47U and 61C are shown in red letters.

The mechanism by which TDP-43 regulates pre-mRNA splicing via its direct or indirect binding to *cis*-elements of introns has been well characterized (5,13–17,58,59). Our present results provide another possible mechanism by which TDP-43 regulates pre-mRNA splicing via controlling site-specific 2′-*O*-methylations of U1 and U2 snRNAs. In canonical pre-mRNA splicing, the removal of introns from pre-mRNA occurs by the dynamic action of the spliceosome, which is formed by the orchestrated assembly of U1, U2, U4/U6 and U5 snRNPs and the other proteins on the consensus sequence regions of pre-mRNA at the 5′ and 3′ splice sites and the branch-site close to the 3′ splice site (Figure 7A) (60). In the first two steps, U1 snRNP assembles on the region at the 5′ splice site and forms the spliceosomal early (E)-complex in assistance with U2 snRNP (61), which results in base-pairing with the region at the branch site to generate the pre-spliceosome (A-complex) (Figure 7A). Given that 2′-*O*-methylation at positions 1, 2, 12 and 19 in the 5′ side region of U2 snRNA is required for the formation of the E-complex (62), our present results suggest that TDP-43 has a role in regulating this step through its ability to control the 2′-*O*-methylation at position 19 in U2 snRNA. Thus, our first proposed mechanism by which TDP-43 regulates pre-mRNA splicing is by controlling the E-complex formation (Figure 7A). Therefore, it will be interesting to know whether loss of function of TDP-43 suppresses the formation of the E-complex or the pre-spliceosome *in vivo* or the other spliceosomal complexes, including those described below.

After these steps, the U4/U6 and U5 tri-snRNPs then assemble on the A-complex to generate the spliceosomal B-complex, which leads to conformational rearrangements of RNA–RNA/RNA–protein/protein–protein interactions to form the activated spliceosome (B*-complex) via dissociation of U1 and U4 snRNPs (Figure 7A). During this rearrangement, the B*-complex is activated by the formation of base-pairs between U2 and U6 snRNAs to catalyze the first trans-esterification at the 5′ end site of the intron and gives rise to the C-complex, which is an intermediate having the 3′ exon—the lariat intron-5′ exon structure within the pre-mRNA. The second trans-esterification step combines the two exons by removing the lariat intron and converts the C-complex to the post-spliceosomal complex, which is composed of the spliced mRNA excised lariat intron and U2/U5/U6 snRNPs. Finally, the U2/U5/U6 snRNPs are recycled for new rounds of splicing after disassembly of the post-spliceosome complex (Figure 7A). During those steps, U2/U5/U6 snRNPs stay in post-B-complex (B*-complex) and, in particular, U2 and U6 snRNAs have critical roles in splicing reactions via their ability to form at least two stable conformations between them under Mg^2+^-dependent dynamic equilibrium; one has a four-helix structure and the other has a three-helix structure (Figure 7B) (63–74. In relation to our present study, post-transcriptional modifications in stem I of U2 snRNA, including 2′-*O*-methylation at position 19, stabilize the four-helix structure and modulate the dynamic equilibrium of the U2 snRNA−U6 snRNA complex (73). Thus, the second possible mechanism we suggest is that TDP-43 regulates the splicing reaction via the ability to maintain the 2′-*O*-methylation at position 19 in stem I of U2 snRNA (Figure 7A and B).

During the splicing reaction, U2 snRNA has two mutually exclusive conformations in the stem II, stem IIa and stem IIc (Figure 7C), which consist of different base-pairings and are interchangeable as a result of the actions of multiple spliceosome components (75,76). The stem IIa conformation is probably present in the spliceosomal A- and B-complexes, whereas the stem IIc conformation is present in the catalytically activated complexes including the B*-complex (Figure 7A) (75,77–81). Stress-inducible pseudouridylations at position 56U and 93U of U2 snRNA are required for the structural equilibrium between stem IIa and stem IIc in yeast cells (82). Because TDP-43 regulates 2′-*O*-methylation at positions 47U and 61C, which are located within stem IIa and stem IIc, respectively (Figure 7C), TDP-43 may also regulate the inter-conversion of those two conformations via its ability to control 2′-*O*-methylation at these positions (Figure 7A and C).

In contrast to U2 snRNA, there has been no report regarding the role of 2′-*O*-methylation at 70A in U1 snRNA function. The crystal structure analysis of the U1A protein−U1hpII oligonucleotide (corresponding to stem II of U1 snRNA) complex has shown that the base at 70A of U1 snRNA stabilizes the complex via its binding to Thr89 and Ser91 of U1A protein (83). Determining whether ribose methylation at 70A affects the binding to U1A protein may clarify whether TDP-43 participates in the formation of a stable U1 snRNA−U1A protein complex.

Although scaRNA2, scaRNA9 and scaRNA7 contain sequences (including that corresponding to mgU2-61) that are predicted to guide or inhibit post-transcriptional modifications in ribosomal RNAs (51,56) (Supplementary Table S7), TDP-43 is not involved in regulating those post-transcriptional modifications, even though depletion of TDP-43 increased the amount and nucleolar accumulation of mgU2-61, as well as the nucleolar localization of scaRNA7 and scaRNA9 (Supplementary Table S7). Those scaRNAs may not participate in the post-transcriptional modification of ribosomal RNAs, or additional regulatory steps may be required for this activity.

Finally, given that TDP-43 maintains the cellular level of CBs and that these levels are reduced in motor neuron diseases such as ALS and spinal muscular atrophy (SMA) (18,19), de-localization of the C/D scaRNAs in CBs may also occur in ALS and SMA. In a cell model causing aggregation of TDP-43, scaRNA7 and scaRNA28 are de-localized to the nucleolar fraction (Figure 4H). Therefore, it is interesting to note that reduced 2′-*O*-methylation may be found in U1 and U2 snRNAs in individuals with ALS and SMA. In general, post-transcriptional modifications, including pseudouridylations and 2′-*O*-methylations in U snRNAs seem to be key regulators of pre-mRNA splicing, and thus of gene expression (49). Pre-mRNA splicing requires the dynamic assembly and orchestrated function of the spliceosome such that each of the U snRNPs binds, changes its conformation and dissociates at the proper site and proper time and in the proper order (60). Site-specific modifications of U snRNAs must be regulated both temporally and spatially during these steps (33,49). TDP-43 may be a key factor that determines the timing of such site-specific modifications. Although the roles of post-transcriptional modifications in the branch point recognition region of U2 snRNA have well been established, roles of the other post-transcriptional modifications of U1 and U2 snRNAs remain largely unknown (84). Current advancement in technologies analyzing post-transcriptional modifications of RNA, such as those including that described in this study, will be useful to elucidate the roles of post-transcriptional modifications of U snRNAs in the regulation of splicing reactions in the context of cellular function.

## Supplementary Material

Supplementary Data
