# Supplementary material for: TDP-43 regulates site-specific 2′-O-methylation of U1 and U2 snRNAs via controlling the Cajal body localization of a subset of C/D scaRNAs
Source: Nucleic Acids Res. 2019 Feb 13;47(5):2487–505. doi: 10.1093/nar/gkz086 (PMC6412121; doi:10.1093/nar/gkz086)
Supplement: Supplementary Data [file gkz086_supplemental_files.zip › Izumikawa et al Supplementary Materials_Rivised.pdf]

Supplementary Materials

TDP-43 regulates site-specific 2'-*O*-methylation of U1 and U2 snRNAs via controlling the Cajal body localization of a subset of C/D scaRNAs

Keiichi Izumikawa[1], Yuko Nobe[2], Hideaki Ishikawa[1], Yoshio Yamauchi[2], Masato Taoka[2], Ko Sato[2], Hiroshi Nakayama[3], Richard J Simpson[1, 4], Toshiaki Isobe[2], and Nobuhiro Takahashi[1*]

[1] Department of Applied Biological Science & Global Innovation Research Organizations, Tokyo University of Agriculture and Technology, 3-5-8 Saiwai-cho, Fuchu, Tokyo 183-8509, Japan

[2] Department of Chemistry, Graduate School of Science, Tokyo Metropolitan University, 1-1 Minami-ohsawa, Hachioji, Tokyo 192-0397, Japan

[3] Biomolecular Characterization Unit, RIKEN Center for Sustainable Resource Science, 2-1, Hirosawa, Wako, Saitama 351-0198, Japan

[4] La Trobe Institute for Molecular Science (LIMS), LIMS Building 1, Room 412 La Trobe University, Melbourne Victoria 3086, Australia

*Corresponding author: Keiichi Izumikawa, Ph.D. and Nobuhiro Takahashi, Ph.D., Department of Applied Biological Science, Tokyo University of Agriculture and Technology, 3-5-8 Saiwai-cho, Fuchu, Tokyo 183-8509, Japan. Tel./Fax: 81-042-367-5703; E-mail: izumic@cc.tuat.ac.jp, ntakahas@cc.tuat.ac.jp

SUPPLEMENTARY FIGURE LEGENDS

Supplementary Figure S1. SILNAS-based post-transcriptional modification analysis of RNase T1 or A–digested U1 and U2 snRNA. (*A*) Extracted ion monitoring of RNase T1–digested fragments of U1 snRNA. U1 snRNA prepared from ncRNA-treated HeLa cells was digested with RNase T1, separated by LC, and monitored at the indicated ion masses. MS spectra of $[ACCCCUGp]^{2-}$ and $[ACCCCU^*Gp]^{2-}$ prepared from ncRNA- and siRNA-treated HeLa cells are shown in the lowest left and right panels, respectively. RNase T1–digested fragments of U1 snRNA prepared from HeLa cells treated with TDP-43 siRNA were similarly monitored (right panel). In those mass chromatograms, the modified fragment CAmCUCCGp was eluted later than the unmodified fragment CACUCCGp, and the corresponding heavy fragment CACUCC*Gp (*G, $^{13}C_{10}$-guanosine) was derived from the reference RNA. The mass windows used for extraction were ±5 ppm. From the peak areas of the light (25.5) and heavy (100) ion and the methylated ion (61.8), the extent of methylation in region 69–75 of U1 snRNA in ncRNA-treated cells was estimated as $[61.8/(61.8 + 25.5)] \times 100 = 70.8\%$, whereas that in siRNA-treated cells was estimated as $[16.3/(78.6 + 16.3)] \times 100 = 17.2\%$. (*B*) Extracted ion monitoring of RNase T1 or RNase A–digested fragments of U2 snRNA containing 2’-*O*-methylation. RNase T1 or RNase A–digested fragments of U2 snRNA prepared from ncRNA-treated HeLa cells were monitored at the indicated ion masses. RNase T1 or RNase A–digested fragments of U2 snRNA prepared from HeLa cells treated with TDP-43 siRNA were similarly monitored. The mass windows used for extraction were ±5 ppm. From the peak areas of the light and heavy (100) ion and the methylated ion, the extent of methylation in each region of U2 snRNA in ncRNA-treated cells was estimated as indicated under each of chromatograms. (*C*) Extracted ion monitoring of RNase T1 or RNase A–digested fragments of U2 containing 2’-*O*-methylation is shown for quantitative analysis upon the knockdown of scaRNA9, 2 or 28 with the corresponding ASO.

Supplementary Figure S2. TDP-43 stabilizes scaRNA2 and suppresses the formation of mgU2-61. (*A*) RNAs prepared from HeLa cells treated with siRNA-1,

siRNA-2 or ncRNA (control) for 96-h for TDP-43 knockdown, were detected by northern blotting with DNA probes shown. The levels of TDP-43 were examined by western blot analysis with anti-TDP-43 antibody. *(B)* The graph shows the relative band intensities of RNAs that was normalized with that of 5S rRNA detected by SYBR gold staining. Mean ± SEM, n = 3–4; *P < 0.05, **P < 0.01, Tukey's test vs ncRNA.

Supplementary Figure S3. TDP-43 maintains the CB localization of UG-rich motif–bearing C/D scaRNAs. (*A-D*) FISH analyses for coilin (a CB marker), B23 (a nucleolar marker) and scaRNA28 (*A*), scaRNA2 (*B*), scaRNA7 (*C*), and scaRNA9 (*D*) with or without TDP-43 knockdown in HeLa cells. After a 48-h treatment of HeLa cells with ncRNA or TDP-43 siRNA, each scaRNA was ectopically expressed for an additional 48 h and detected with Cy3-labeled DNA probes (red) complementary to the corresponding scaRNA. Coilin and B23 were detected with mouse monoclonal anti-coilin and mouse monoclonal anti-B23 and FITC-conjugated anti–mouse IgG (green). Bars indicate 5 μm. FITC and Cy3 signals were merged (Merge); DAPI for DNA staining was overlaid with the merged image. (*E*) Immunocytochemical analyses for coilin and TDP-43 with or without TDP-43 knockdown in HeLa cells. After a 96-h treatment of HeLa cells with ncRNA or TDP-43 siRNA, TDP-43 or coilin were detected with rabbit polyclonal anti-TDP-43 IgG and FITC-conjugated anti–rabbit IgG (green), or mouse monoclonal anti-coilin IgG and Cy3-conjugated anti–mouse IgG (red), respectively. Bars indicate 5 μm. FITC and Cy3 signals were merged (Merge); DAPI for DNA staining was overlaid with the merged image. The number of coilin-staining dots per cell was counted visually. N = 227 cells for ncRNA, 213 cells for siRNA-1, and 224 cells for siRNA-2. The graph (upper) shows the distribution of the number of coilin-staining dots per cell. The graph (bottom) shows the average number of coilin-staining dots per cell. Mean ± SEM; **P < 0.01, tukey-kramer's test. *(F)* FISH analyses for TDP-43, and scaRNA28 (upper) or scaRNA7 (lower) with or without doxycycline in TDP-12xQ/N expressing T-REx 293 cells (TDP-12xQ/N cells). After a 48-h treatment of TDP-12xQ/N expressing T-REx 293 cells with or without doxycycline, scaRNA28 or scaRNA7 was ectopically expressed for an additional 48 h and detected with Cy3-labeled

DNA probes (red) complementary to the corresponding scaRNA. TDP-43 were detected with rabbit polyclonal anti-TDP-43 and FITC-conjugated anti–rabbit IgG (green). Bars indicate 5 μm. FITC and Cy3 signals were merged (Merge); DAPI for DNA staining was overlaid with the merged image.

Supplementary Figure S4. WDR binds TDP-43 independently of RNA but does not affect CB localization of UG-rich motif–bearing C/D scaRNAs. (*A*) DAP-TDP-43 immunoprecipitated using anti-FLAG M2 agarose was treated with (+) or without (–) RNase A at 4°C (–) or 37°C (+). DAP-TDP-43 and WDR79 were detected using streptavidin-HRP (biotin) and anti-WDR79, respectively. Input, 10 μg of cell lysate and 4 μg of RNA extracted from cell lysates. (*B*) Immunoprecipitation of DAP-TDP-43 (WT) or its deletion mutants using anti-FLAG from DAP-TDP-43 or each deletion mutant–expressing T-REx 293 cells after treatment with doxycycline for 24 h. Proteins were detected by western blot analysis with anti-WDR79 or streptavidin-HRP. Input, 10 μg of cell lysate. (*C*) Immunoprecipitation of endogenous WDR79 using anti-WDR79 from cell lysates of 293T cells was analyzed by western blotting with anti-TDP-43 and anti-WDR79. Nonspecific rabbit IgG (IgG) was used as a control. Input, 10 μg of cell lysate. (*D*) HEF-WDR79 was immunoprecipitated using anti-FLAG from HEF-WDR79–expressing T-REx 293 cells after treatment with doxycycline for 24-h. Western blot analysis (IB) with antibodies indicated and northern blot analysis (NB) with DNA probes indicated on the right are shown. HEF-WDR79 was detected with anti-FLAG. Input, 10 μg of cell lysate and 4 μg of RNA extracted from cell lysates. T-REx 293 cells (T-REx) were used as a control. (*E*) Immunocytochemical analyses for WDR79 and coilin with or without WDR79 knockdown in HeLa cells. After a 96-h treatment of HeLa cells with ncRNA or WDR79 siRNA, WDR79 or coilin were detected with rabbit polyclonal anti-WDR79 IgG and FITC-conjugated anti–rabbit IgG (green), or mouse monoclonal anti-coilin IgG and Cy3-conjugated anti–mouse IgG (red), respectively. Bars indicate 5 μm. FITC and Cy3 signals were merged (Merge); DAPI for DNA staining was overlaid with the merged image. (*F*) FISH analyses of WDR79 and scaRNA2 (upper), 7 (middle), or 9 (lower) with or without WDR79 knockdown in HeLa cells. After a 48-h treatment of HeLa cells with ncRNA or

WDR79 siRNA, scaRNA2, 7, or 9 was ectopically expressed for an additional 48 h and detected with Cy3-labeled corresponding scaRNA DNA probe (red). WDR79 was detected with rabbit polyclonal anti-WDR79 and FITC-conjugated anti–rabbit IgG (green). Bars indicate 5 μm. FITC and Cy3 signals were merged (Merge); DAPI for DNA staining was overlaid with the merged image.

Supplementary Figure S1

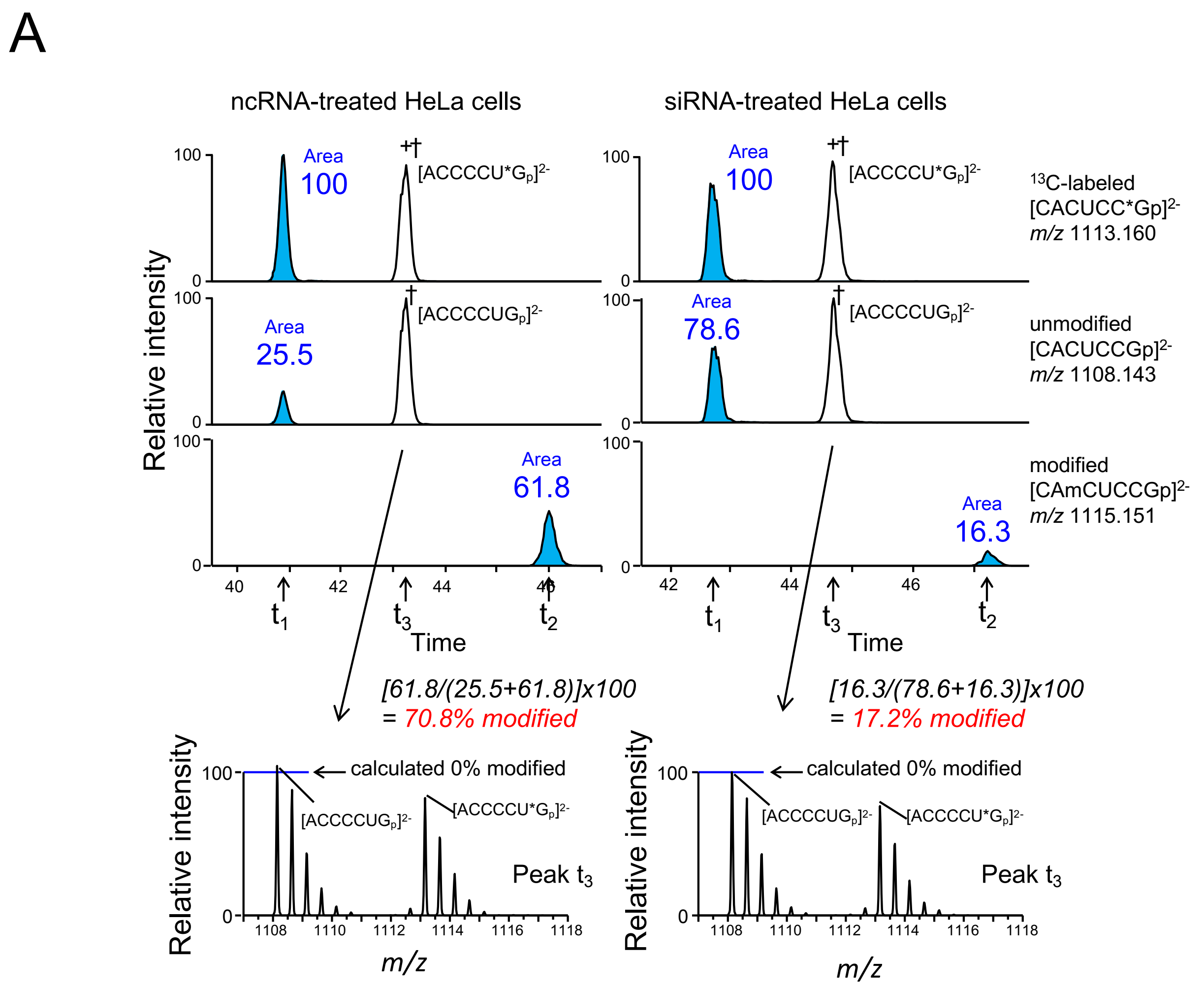

Supplementary Figure S1

B

U2-19Gm

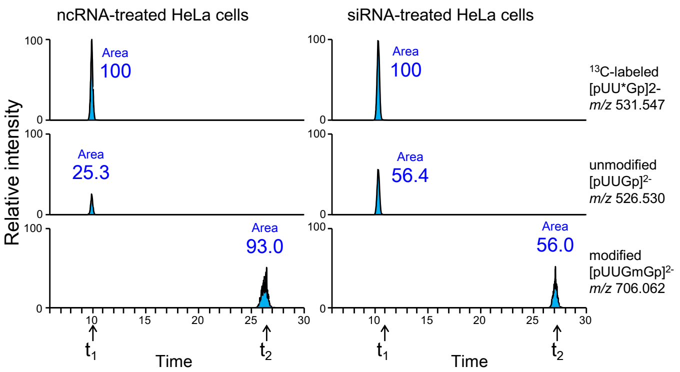

Ratio of UUGmG can not be calculated by peak area.

U2-25Gm

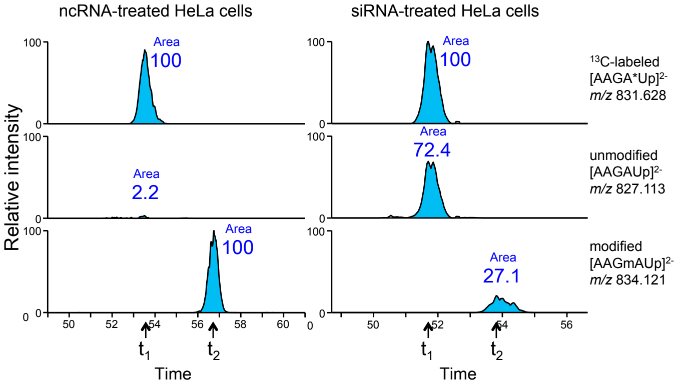

[100/(2.2+100)] x100
= *97.9% modified*

[27.1/(72.4+27.1)]x100
= *27.2% modified*

U2-47Um

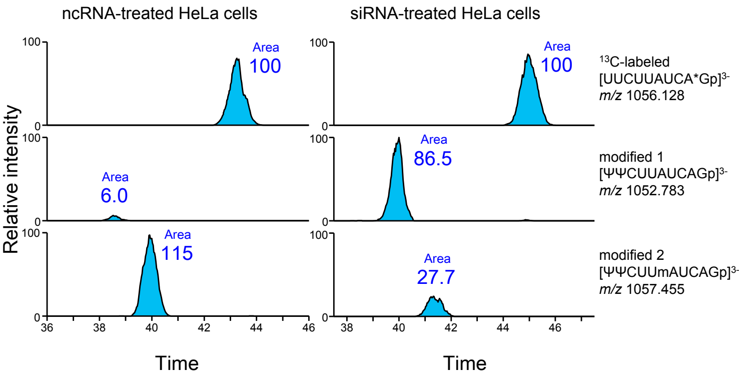

[115/(6+115)]x100
= *95.0% modified*

[27.7/(86.5+27.2)]x100
= *24.3% modified*

U2-61Cm

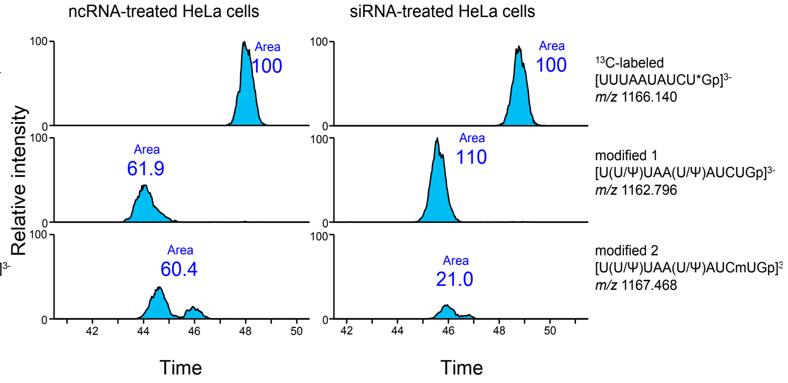

[60.4/(61.9+60.4)]x100
= *49.4% modified*

[21/(21+110)]x100
= *16.0% modified*

U2-40Cm

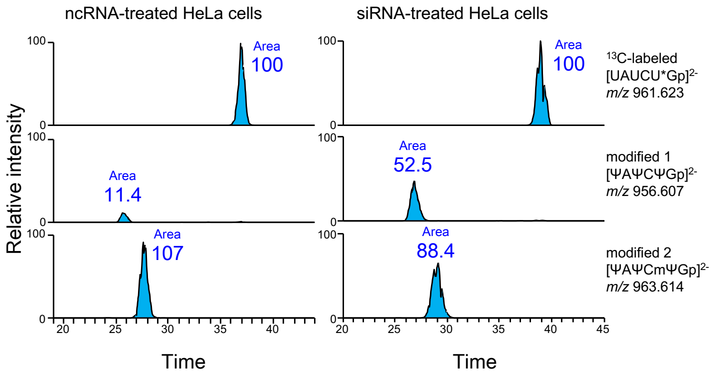

[107/(11.4+107)]x100
= *90.4% modified*

[88.4/(52.5+88.4)]x100
= *62.7% modified*

Supplementary Figure S1

U2-19Gm

C

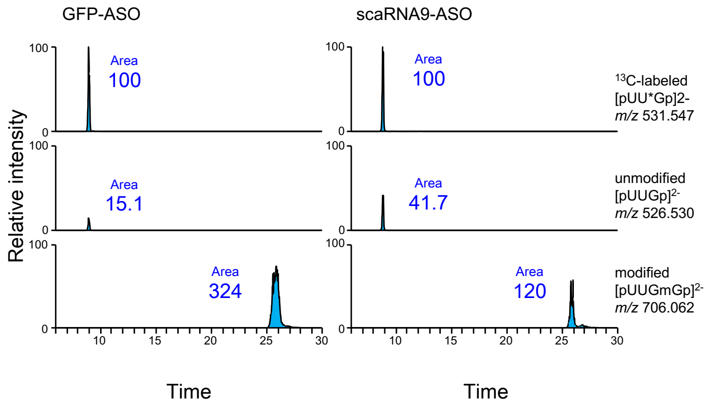

U2-25Gm

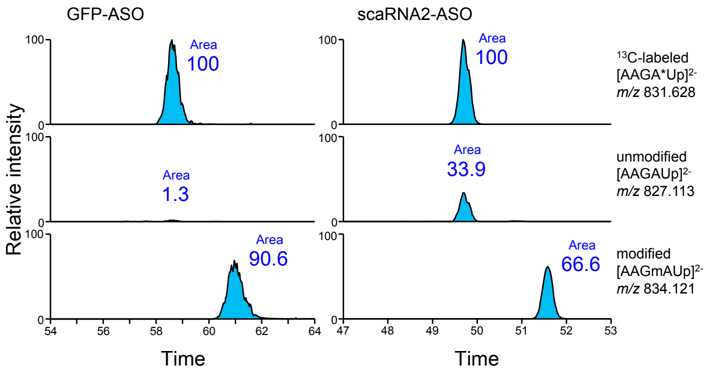

U2-61Cm

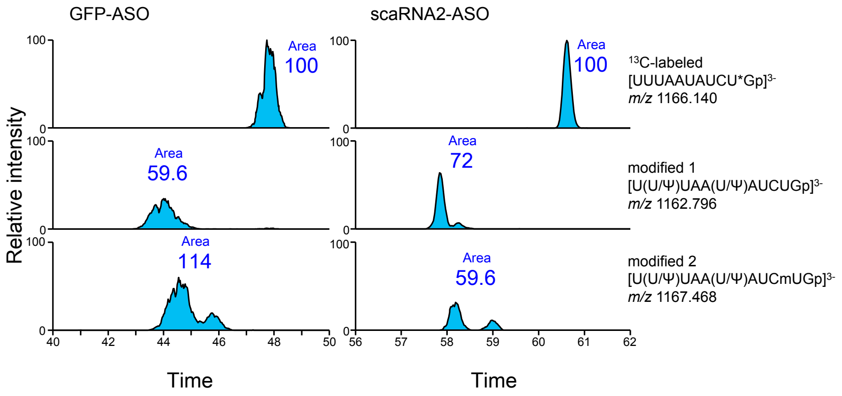

U2-47Um

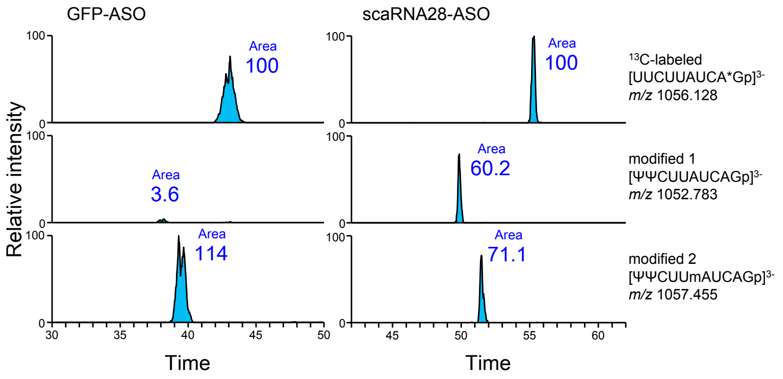

Supplementary Figure S2

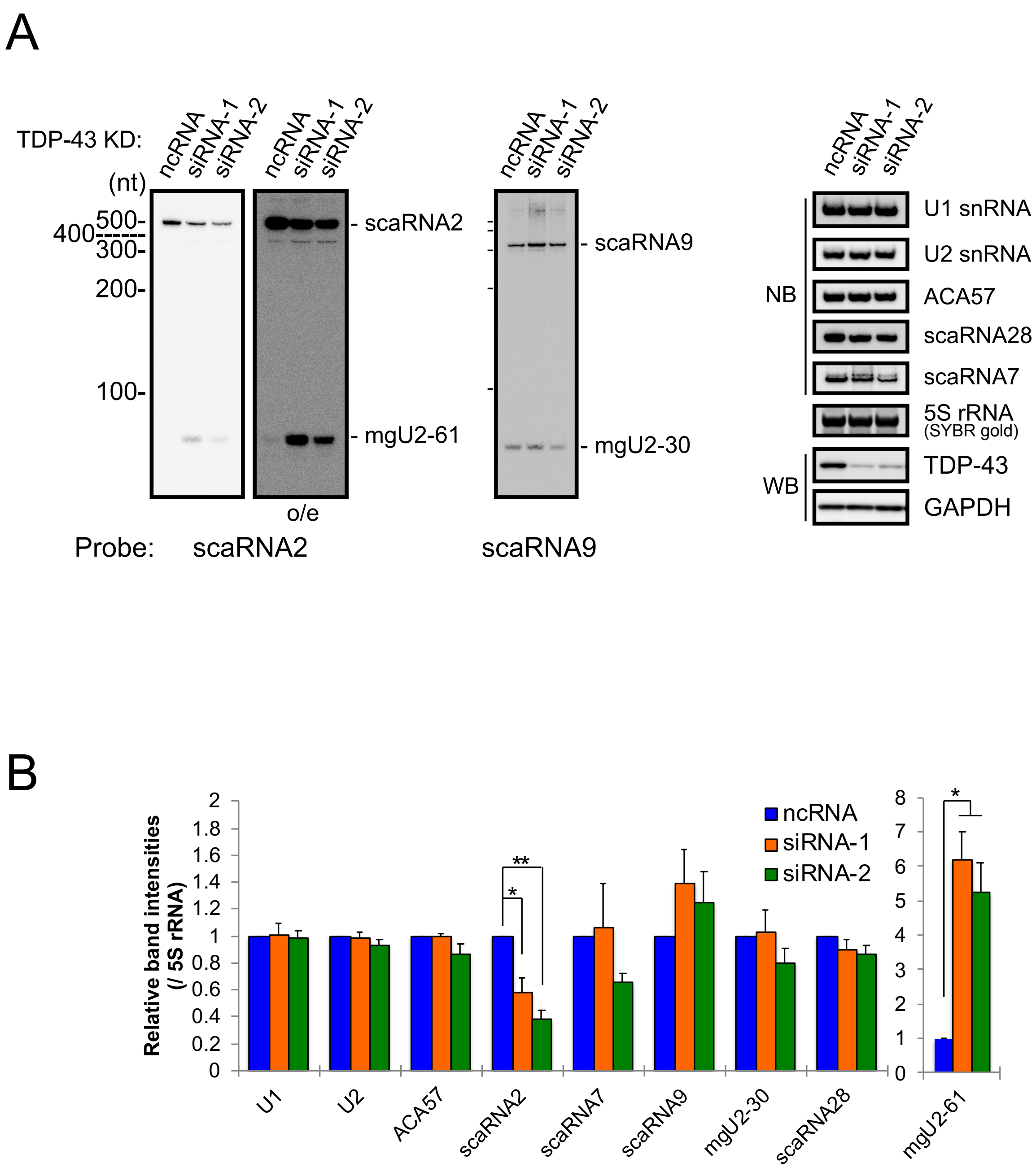

Supplementary Figure S3

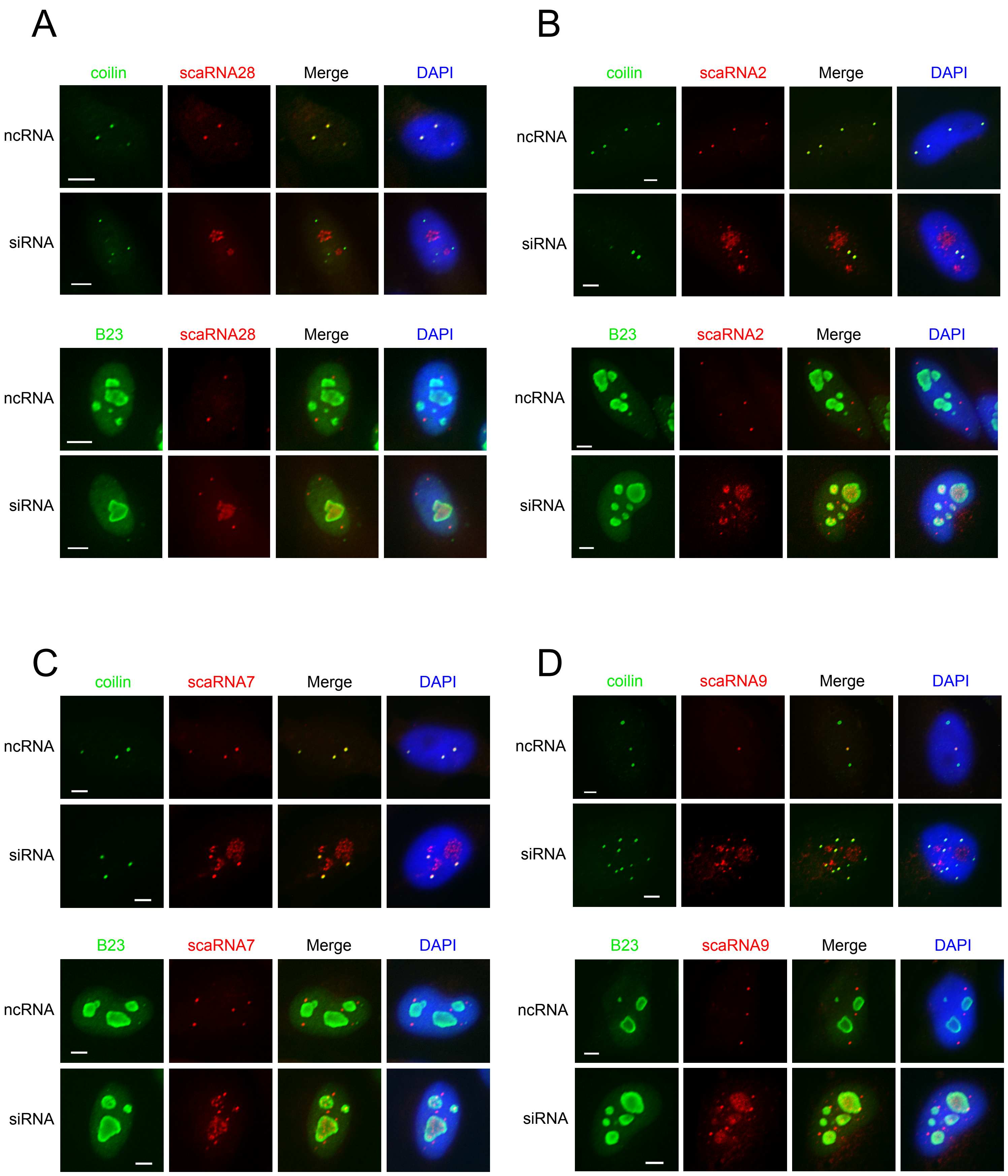

Supplementary Figure S3

E

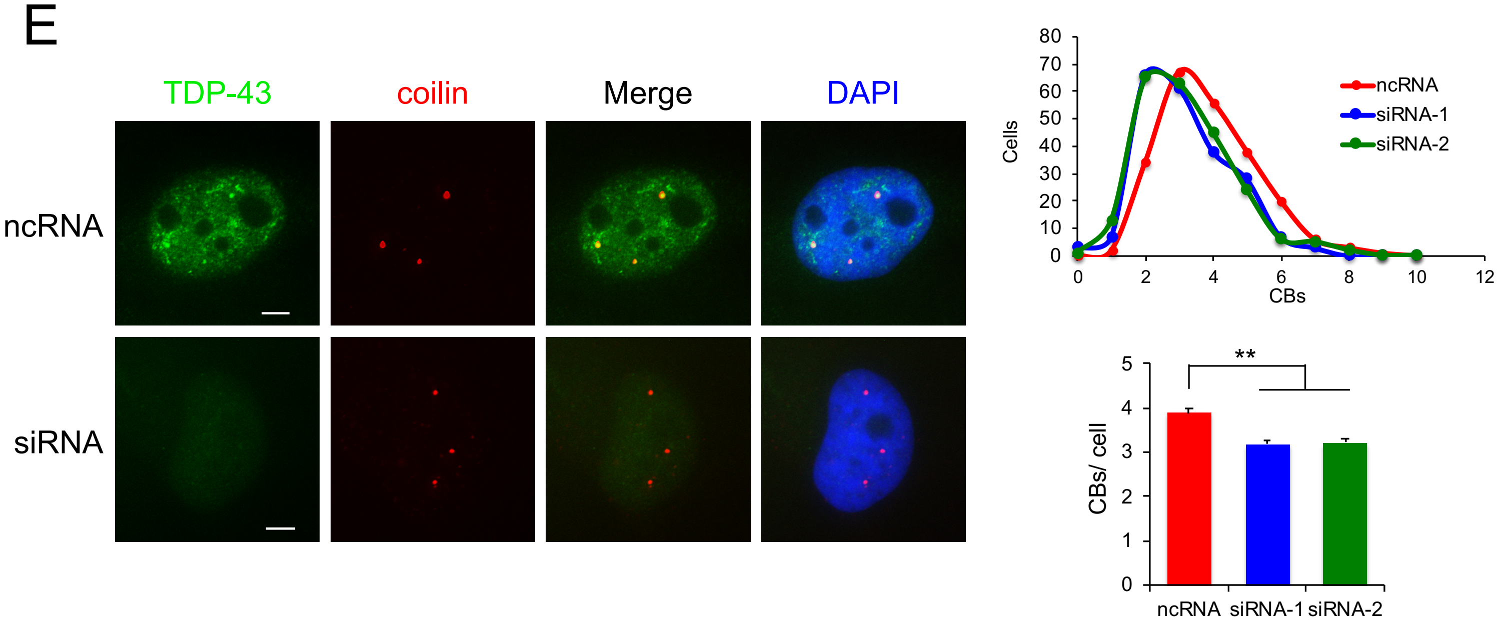

F

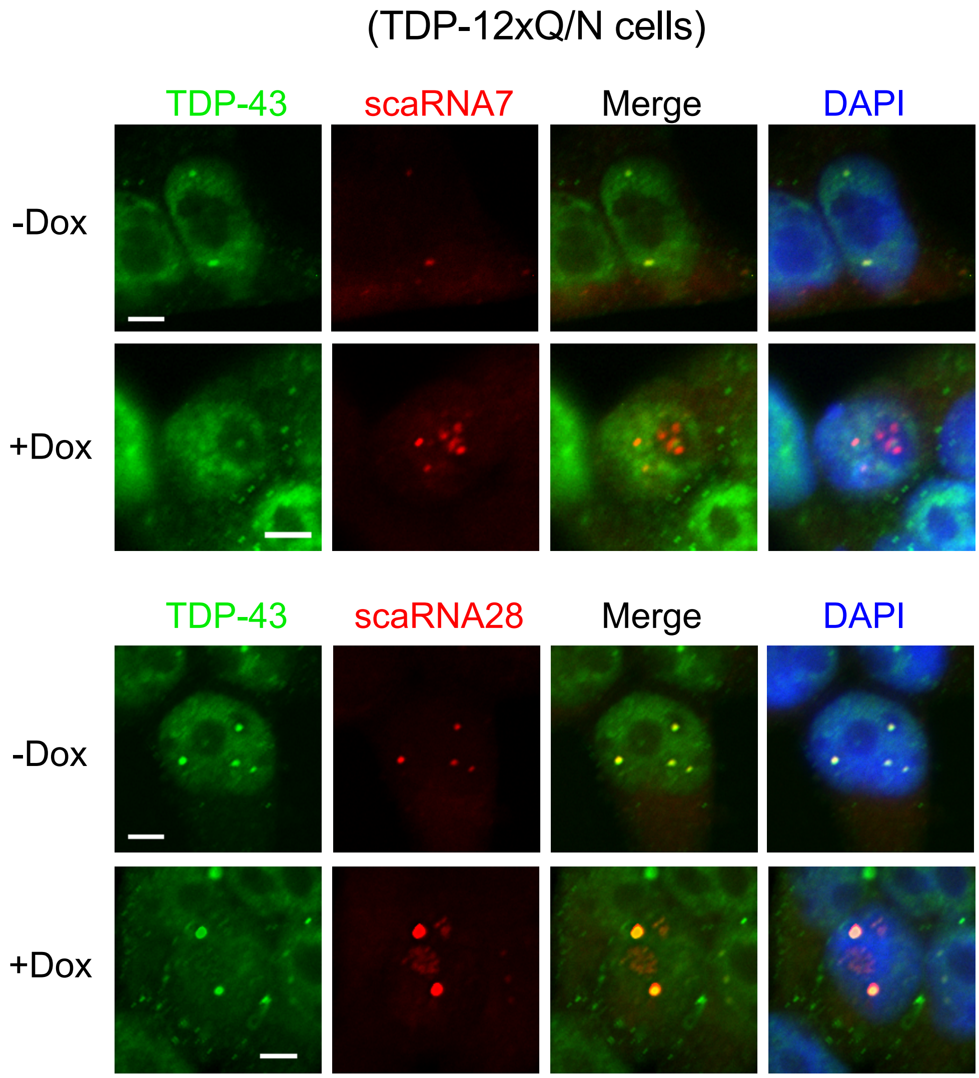

Supplementary Figure S4

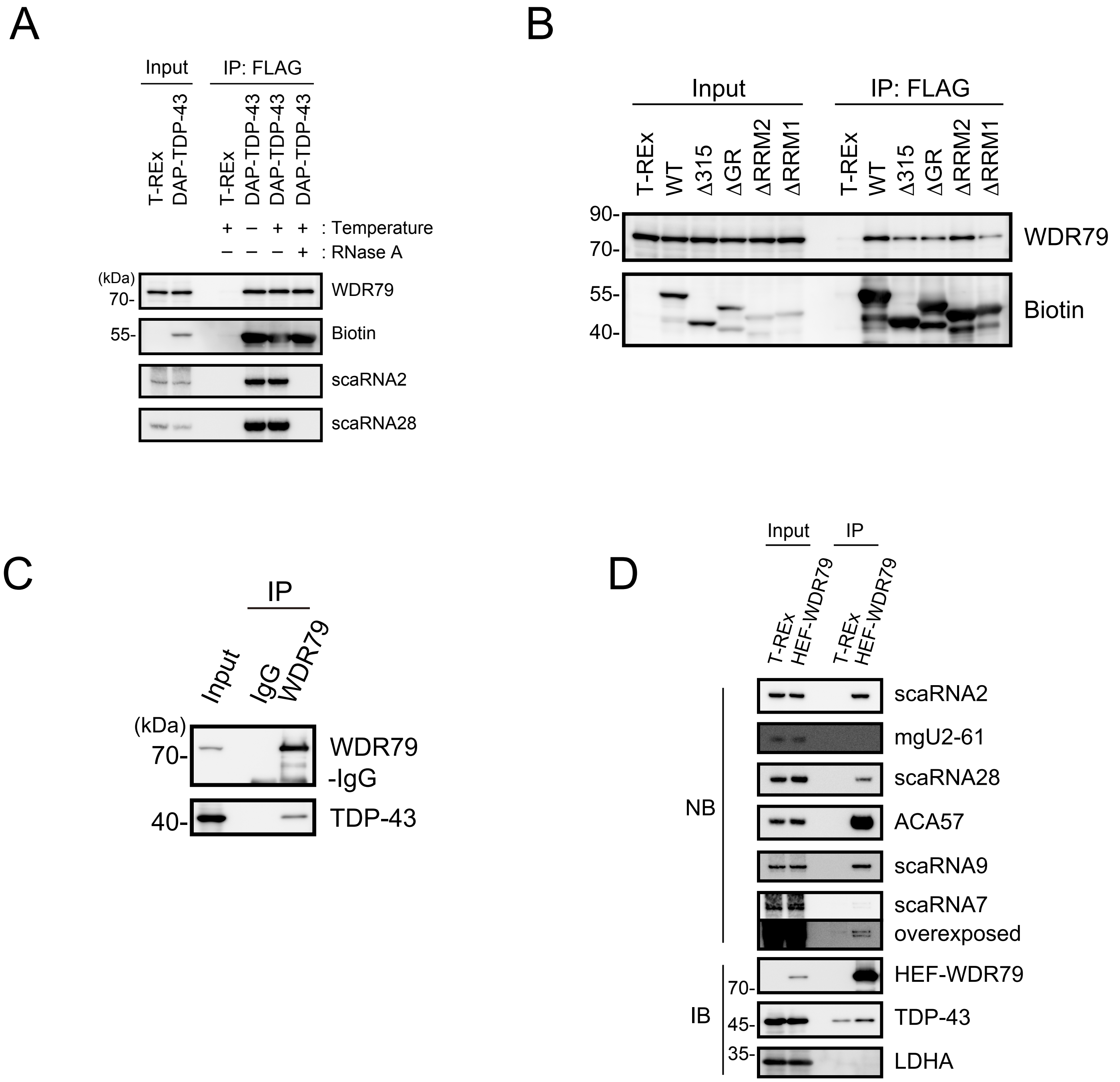

Supplementary Figure S4

E

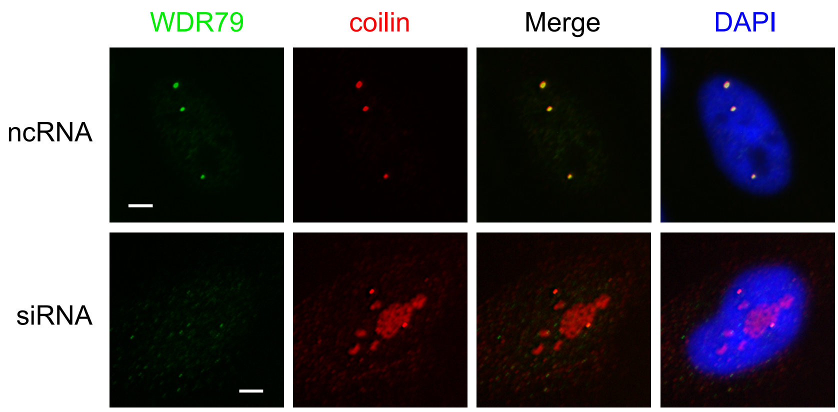

F

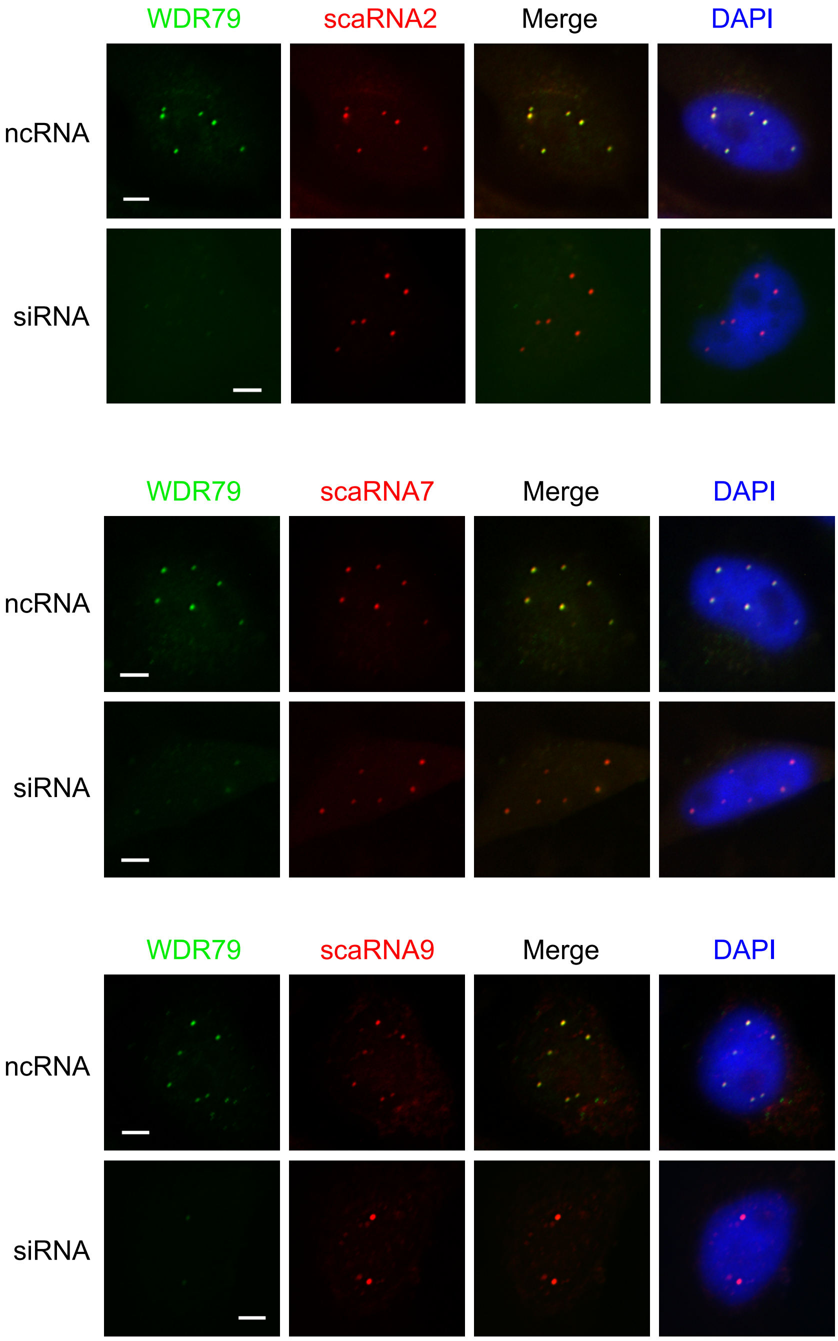

Supplementary Table information.

Supplementary Table S3. Supplementary_Table_S3.xlsx

Supplementary Table S5. Supplementary_Table_S5.xlsx

Supplementary Table S1. List of antibodies used in this study

Antibody	Host/Class	Supplier	Product code	Used for
anti-FLAG	mouse / monoclonal	Sigma-Aldrich	F3165	WB, ICS
anti-FLAG	rabbit / polyclonal	Sigma-Aldrich	F7425	ICS
anti-nonspecific rabbit IgG	rabbit / polyclonal	Abcam (Cambridge, UK)	ab46540	IP
anti-GAPDH	mouse / monoclonal	Ambion	AM4300	WB
anti-TDP-43	mouse / monoclonal	Proteintech	60019-2-Ig	WB
anti-TDP-43	rabbit / polyclonal	Proteintech	10782-2-AP	ICS, WB
anti-WDR79	rabbit / polyclonal	Bethyl Laboratories	A301-442A	WB, ICS, IP
anti-coilin	mouse / monoclonal	Sigma-Aldrich	C1862	ICS
anti-B23/Nucleophosmin	mouse / monoclonal	ZYMED	Fc-61991	ICS
anti-LDHA	rabbit / polyclonal	Proteintech	21799-1-AP	WB
anti-rabbit IgG, FITC-conjugated		American Qualex	A602FS	ICS
anti-mouse IgG, FITC-conjugated		KPL	02-18-06	ICS
anti-mouse IgG, Cy3-conjugated		Sigma-Aldrich	C2181	ICS
anti-mouse IgG, HRP-conjugated		Cell Signaling Technology	#7076	ICS
anti-rabbit IgG, HRP-conjugated		Cell Signaling Technology	#7074	WB
anti-goat IgG, HRP-conjugated		Sigma-Aldrich	A5420	WB

Supplementary Table S2. List of siRNA, primer, probe and antisence oligonucleotide used

siRNA	Sequence	Used for	Others
ncRNA	ncRNA Silencer Select Negative Control No. 1 siRNA Silencer Select siRNA	siRNA	Stealth siRNA
si TDP-43 #1 (siRNA-1)-sense	UUAAGAUCUUUCUUGACCUGCACCA	siRNA	Stealth siRNA
si TDP-43 #1 (siRNA-1)-antisense	UGGUGCAGGUCAAGAAAGAUCUUAA	siRNA	Stealth siRNA
si TDP-43 #2 (siRNA-2)-sense	GACAGAUGCUUCAUCAGCAGUGAAA	siRNA	Stealth siRNA
si TDP-43 #2 (siRNA-2)-antisense	UUUCACUGCUGAUGAAGCAUCUGUC	siRNA	Stealth siRNA
ncRNA	Silencer Select Negative Control No. 1 siRNA	siRNA	Silencer Select siRNA
si WDR79-sense	CCUCUGCUUUCAUCCCGAUtt	siRNA	Silencer Select siRNA
si WDR79-antisense	AUCGGGAUGAAAGCAGAGGtg	siRNA	Silencer Select siRNA

Primer Name	Sequence 5'-->	Target
TRRAP-for	TATAGGATCCATGGCGTTTGTTGCAACACAGGGGGCCACG	DAP-TRRAP(Ex2-Int2-Ex3)
TRRAP-rev	ATGATGCAAGAAGTTAGTGAAAATTTTGAGTGACTCGAGTATA	
WDR79-for	TATAAAGCTTATGAAGACTTTGGAGACTCAACCG	HEF-WDR79
WDR79-rev	TATACTCGAGTTATATCAGCTCACCCACACCTCC	
T7-scaRNA28-for	CTAATACGACTCACTATAGGGAGAGCAAAGTGATGAGTAATACTGGCTG	Template DNA for in vitro transcription
T7-scaRNA28-rev	GCAATCAGATCTTATCAGTTTGACTGTC	
T7-scaRNA2-for	CTAATACGACTCACTATAGGGAGAGTTTTAGGGAGGGAGAGCGGCC	Template DNA for in vitro transcription
T7-scaRNA2-rev	CCAGATCAGAATCGCCTCGATA	
T7-scaRNA9-for	CTAATACGACTCACTATAGGGAGACTTTCTGAGATCTGCTTTTAGT	Template DNA for in vitro transcription
T7-scaRNA9-rev	TGAGCTCAGGTCAAGTGTAGAA	
T7-scaRNA7-for	CTAATACGACTCACTATAGGGAGATGGAAATGATGAAATAGAGATA	Template DNA for in vitro transcription
T7-scaRNA7-rev	TTGGATCAGATCACAGGACCAT	
GAPDH-for	CCTCCAAGGAGTAAGACCCC	qPCR for GAPDH
GAPDH-rev	TGTGAGGAGGGGAGATTCAG	
TDP-43-for	GCATGTTCAA AACGGAAACCATTG	qPCR for TDP-43
TDP-43-rev	AACACTGTGA CACCATGATCTCC	
pBS-hU1A-F	gctctcgagGATACTTACCTGGCAGGGGA	Used for PCR-amplification of the U1 snRNA cDNA
pBS-hU1A-R	gacgaattcCAGGGGAAAGCGCGAACGCA	
005_U2_InF_F	GTTTAAACTTAAGCTTATCGCTTCTCGGCCTTTTG	Used for PCR-amplification of the U2 snRNA cDNA
006_U2_InF_R	GCCCTCTAGACTCGAGTGGTGCACCGTTCCTGGA	
107_U2-1-chimera*	AmUmCmUmUmAmGmCmCmAmAm(AAGG)CmCmGmAmGmAmAmGm	Used for RNase H clevage of U2 snRNA

Probe	Sequnence	Used for	Region of hybridyzation
scaRNA28-BT	GGGCTCCAGCCAGTATTACTCATCACTTTG	Northern blot	2-31 in scaRNA28
scaRNA2-BT	TCGTCTATCTGATCAATTCATCACTTCT	Northern blot	345-372 in scaRNA2
scaRNA9-BT	AGCTCAGGTCAAGTGTAGAAACCATCATAG	Northern blot	322-351 in scaRNA9
scaRNA7-BT	CTAGTTAGGTGAGCTGTTCTTGGG	Northern blot	250-273 in scaRNA7
scaRNA17-BT	CCTCAGTCTGTTCTCAGAACATACTCC	Northern blot	392-418 in scaRNA17
ACA57-BT	AATTGTCCTCTCGTGCCTTTAAGAGCC	Northern blot	111-132 in ACA57
scaRNA8-BT	GTCTGCCCCGTATCTGTCCGTTACGATT	Northern blot	100-127 in scaRNA8
U85-BT	ATACCCTGAACATGCCCTGTTCCT	Northern blot	226-249 in U85 scaRNA
U3-BT	ATCATCAATGGCTGACGGCAGTTG	Northern blot	141-164 in U3 snoRNA
tRNA(Met)-BT	TAGCAGAGGATGGTTTCGATCCATCGA	Northern blot	46-72 in tRNA(Met)
mt-tRNA(Asn)-BT	CTAGACCAATGGGACTTAAACCCACAAACACTTA	Northern blot	40-73 in mt-tRNA(Asn)
U2-FITC	CTACACTTGATCTTAGCCAAAAGGCCGAGAAGC	FISH	4-36 in U2 snRNA
U2-Cy3	CTACACTTGATCTTAGCCAAAAGGCCGAGAAGC	FISH	4-36 in U2 snRNA
scaRNA2-Cy3	TCGTCTATCTGATCAATTCATCACTTCT	FISH	345-372 in scaRNA2
scaRNA9-Cy3	GAGCTCAGGTCAAGTGTAGAAACCATCATAG	FISH	322-352 in scaRNA9
scaRNA28-Cy3	GGGCTCCAGCCAGTATTACTCATCACTTTG	FISH	2-31 in scaRNA28
scaRNA7-Cy3	TACACCCAATTATCTCTATTTCATCATTTCCAT	FISH	1-31 in scaRNA7

Anti sense oligonucleotide*	Sequnence	Used for	Region of hybridyzation
scaRNA28-ASO	UmCmUmUmAm(TCAGTTTGAC)UmGmUmCmAm	Knockdown of scaRNA	159-178 in scaRNA28
scaRN7-ASO	AmUmGmCmAm(CTCCAATATC)AmGmCmAmUm	Knockdown of scaRNA	172-191 in scaRNA7
scaRNA2-ASO	UmAmAmGmAm(TCAAAGTGTA)AmGmCmGmGm	Knockdown of scaRNA	166-185 in scaRNA2
scaRNA9-ASO	GmUmCmAmAm(GTGTAGAAAC)CmAmUmCmAm	Knockdown of scaRNA	325-344 in scaRNA9
GFP-ASO	UmCmAmCmCm(TTCACCCTCT)CmCmAmCmUm	Knockdown of scaRNA	

*; Nm refers to 2'-O-methyl ribonucleotide. Deoxyribonucleotides are indicated in parentheses.

Supplementary Table S4. List of scaRNAs

Name1	Name2	Target	C/D or H/ACA	Length	Motif
scaRNA1	ACA35	U2-89Ψ	H/ACA	166	
scaRNA2	mgU2-25/61, HBII-382	U2-11Gm, U2-25Gm<br>U2-61Cm	C/D	420	UG-rich
scaRNA3	HBI-100	U6-40Ψ	H/ACA	144	
scaRNA4	ACA26	U2-41Ψ, U2-39Ψ	H/ACA	129	
scaRNA5	U87	U5-41Um, U4-65Am	C/D, H/ACA	277	CAB
scaRNA6	U88	U5-41Um	C/D, H/ACA	266	CAB
scaRNA7	U90	U1-70mA	C/D	330	UG-rich
scaRNA8	U92	U2-34Ψ, U2-44Ψ	H/ACA	131	CAB x2
scaRNA9	mgU2-19/30	U2-19Gm, U2-30Am	C/D	353	UG-rich
scaRNA9L	pseudogene		C/D	348	UG-rich
scaRNA10	U85	U5-45Cm, U5-46Ψ	C/D, H/ACA	330	CAB x2
scaRNA11	ACA57	U5-43Ψ	H/ACA	137	CAB x2
scaRNA12	U89		C/D, H/ACA	270	CAB
scaRNA13	U93	U5-53Ψ, U2-54Ψ	H/ACA	275	CAB x3
scaRNA14	U100	U2-7Ψ	H/ACA	140	
scaRNA15	ACA45	U2-37Ψ	H/ACA	127	
scaRNA16	ACA47	U1-5Ψ	H/ACA	187	CAB
scaRNA17	mgU12-22/mgU4-8, U91	U12-22Gm, U4-8Cm	C/D	421	
scaRNA18	U109	U1-6Ψ	H/ACA	133	CAB
scaRNA19	hTR/TERC	telomeres	H/ACA	451	CAB
scaRNA20	ACA66	U12-28Ψ	H/ACA	140	
scaRNA21	ACA68	U6atac-19Ψ, U12-19Ψ	C/D, H/ACA	138	
scaRNA22	ACA11		H/ACA	132	
scaRNA23	ACA12	U6-40Ψ	H/ACA	130	
scaRNA26A			H/ACA	148	
scaRNA26B			H/ACA	149	
scaRNA27			H/ACA	126	
scaRNA28	mgU2-47	U2-47Um	C/D	187	UG-rich

Prepared based on Hüttenhofer et al. 2001; Darzacq et al. 2002; Tycowski et al. 2009; Karijolich and Yu 2014; Bohnsack and Sloan 2018; Meier 2017. Predicted scaRNAs by bioinformatic analysis are listed under Name1. Alternative names are also given under Name2 if they are known. Target sites of the post-transcriptional modifications (Ψ; pseudourydylation, m; 2'-O-methylation) in U snRNA (U1, U2, U4, U5, U6, U6atac, or U12 snRNA) are given for each of scaRNAs under target. Types [C/D scaRNA that guides methylation (C/D) and/or H/ACA scaRNA that guides pseudourydylation (H/ACA)], lengths (nt) of scaRNAs are given. When scaRNA has WDR79 binding motif (CAB) or TDP-43 binding motif (UG-rich), it is given for the corresponding scaRNA.

Supplementary Table S6. Quantification of site-specific methylation (293T cells)

Modified nucleotide	Modified oligonucleotide	293T (% modification)[a]			Guide scaRNA (predicted)	Quantitative methods
**U1 snRNA**		**ncRNA**	**siRNA-1**	**siRNA-2**		
Cap (1Am, 2Um)	TMG-AmUmACYYACCUG	100 ± 0	100 ± 0	100 ± 0	scaRNA16, 18	Peak area
70Am	CAmCUCCG	82.1 ± 1.6	48.3 ± 4.6**	39.6 ± 2.2**	scaRNA7	SILNAS
70Am	CAmCUCCG	79.9 ± 1.6	51 ± 6.5	43.2 ± 7.8*	scaRNA7	Peak area
**U2 snaRNA**						
Cap (1Am, 2Um)	TMG-AmUmC	99.0 ± 0.1	98.9 ± 0.2	99.1 ± 0.1	-	Peak area
11Gm	GmGmC + GmGC	96.6 ± 1.5	97.1 ± 0.7	97.8 ± 0.4	scaRNA2	Peak area
12Gm	GmGmC + GGmC	96.7 ± 1.4	97.2 ± 0.5	97.9 ± 0.4	unknown	Peak area
19Gm	UUGmG	92.9 ± 0.4	81.1 ± 2.3	82.3 ± 3.6	scaRNA9	SILNAS
25Gm	AAGmAU	98.7 ± 0.5	69.8 ± 4.2*	75.9 ± 4.1*	scaRNA2	SILNAS
25Gm	AAGmAU	99 ± 0.6	70.3 ± 4.3*	75.9 ± 3.4*	scaRNA2	Peak area
30Am	Am6AmGU + AAmGU	93 ± 1.7	93.2 ± 1.3	93.5 ± 0.3	scaRNA9	Peak area
34Ψ	ΨAG	78.1 ± 2.8	75 ± 3.4	77.6 ± 1.8	scaRNA8	SILNAS
34Ψ	ΨAG	77.4 ± 4.7	77.6 ± 3.5	75.9 ± 5.6	scaRNA8	Peak area
40Cm	YAYCmYG	98.5 ± 0.5	87.7 ± 3.9	83.1 ± 6.4	unknown	Peak area
47Um	YYCUUmAUCAG	97.9 ± 0.8	50.8 ± 4.4**	47.3 ± 6.4**	scaRNA28	Peak area
61Cm	UYUAAYAUCmUG	83 ± 1.3	38.4 ± 3.7**	39.5 ± 4.8**	scaRNA2	Peak area

m; methy group, Y; U or pseudouridine (Ψ), TMG; 2,2,7-trimethylguanosine (m3G).

a: Values are obtained as mean ± SEM (n = 3). * $P < 0.05$; ** $P < 0.01$ (Tukey's test; vs ncRNA).

TDP-43 mRNA levels (per GAPDH mRNA); ncRNA (1.000 ± 0) : siRNA-1 (0.455 ± 0.077) : siRNA-2 (0.466 ± 0.020).

In the case of analysis U2-cap, Gm11, Gm12, and Gm19; ncRNA (1.000 ± 0), siRNA-1 (0.475 ± 0.058), siRNA-2 (0.504 ± 0.018).

Supplementary Table S7. List of scaRNAs that are expected to guide post-transcriptional modifications in rRNAs

scaRNA	Fragment	Binding	rRNA-mod.site (Poole et al. 2017, Jorhani et al. 2016)	% of mod. (Taoka et al, 2018)	Effect by TDP-43 KD
scaRNA2	mgU2-61	HBII-82B	28S-3923Gm	80	no change
scaRNA9	mgU2-19	ACA61	28S-2495Ψ	92	no change
	mgU2-30	ACA37	28S-4643Ψ	39	no change
	mgU2-30	U20	18S-1804Um	86	no change
scaRNA7			5.8S-75Gm	87	no change

The scaRNA-guided post-transcriptional modifications (PTMs) of rRNA are listed. A region responsible for the guide of the PTM in each scaRNAs is given; e.g. mgU2-61, mgU2-19 etc. PTMs reported by Poole et al. (Poole et al. 2017) is shown. The percentage (%) of PTM at each site of rRNA reported by Taoka et al. is also shown.
